# Are Quasi-Steady-State Approximated Models Suitable for Quantifying Intrinsic Noise Accurately?

**DOI:** 10.1371/journal.pone.0136668

**Published:** 2015-09-01

**Authors:** Dola Sengupta, Sandip Kar

**Affiliations:** Department of Chemistry, IIT Bombay, Powai, Mumbai - 400076, India; Fondazione Edmund Mach, Research and Innovation Centre, ITALY

## Abstract

Large gene regulatory networks (GRN) are often modeled with quasi-steady-state approximation (QSSA) to reduce the huge computational time required for intrinsic noise quantification using Gillespie stochastic simulation algorithm (SSA). However, the question still remains whether the stochastic QSSA model measures the intrinsic noise as accurately as the SSA performed for a detailed mechanistic model or not? To address this issue, we have constructed mechanistic and QSSA models for few frequently observed GRNs exhibiting switching behavior and performed stochastic simulations with them. Our results strongly suggest that the performance of a stochastic QSSA model in comparison to SSA performed for a mechanistic model critically relies on the absolute values of the mRNA and protein half-lives involved in the corresponding GRN. The extent of accuracy level achieved by the stochastic QSSA model calculations will depend on the level of bursting frequency generated due to the absolute value of the half-life of either mRNA or protein or for both the species. For the GRNs considered, the stochastic QSSA quantifies the intrinsic noise at the protein level with greater accuracy and for larger combinations of half-life values of mRNA and protein, whereas in case of mRNA the satisfactory accuracy level can only be reached for limited combinations of absolute values of half-lives. Further, we have clearly demonstrated that the abundance levels of mRNA and protein hardly matter for such comparison between QSSA and mechanistic models. Based on our findings, we conclude that QSSA model can be a good choice for evaluating intrinsic noise for other GRNs as well, provided we make a rational choice based on experimental half-life values available in literature.

## Introduction

Most of the events in and around the biological cell are inherently noisy. The sources of noise in the biological systems are diverse and can be mainly classified into two different classes [1–5], namely intrinsic and extrinsic noise. Recently, there were a number of experimental findings [6–11], which revealed that intrinsic and extrinsic noise levels in a particular cell can dictate its ultimate cell fate to a great extent. The intrinsic noise is mainly classified as the molecular noise that is present in any cell type because of low copy numbers of mRNA molecules corresponding to any particular protein [1,3,12,13]. Further, it is well established [14,15] that the coefficient of variation (CV) of the distribution of a particular protein not only depends on the average number of mRNA and protein molecules but also relies on the absolute values of the half-lives of the corresponding mRNA and protein molecules. Gillespie’s stochastic simulation algorithm (SSA) [16] provides fundamentally the most accurate and correct description of the intrinsic noise for a biological network if all the terms in the corresponding model are based on mass action kinetics. This means as the biological network grows in size and complexities, it will be computationally highly time consuming to simulate every reaction event using SSA. To overcome this challenge, different kinds of efforts had been made to avoid continuously simulating the reactions that happen in a faster time scale in case of SSA [15,17–19]. Quasi-steady-state approximation (QSSA) is one of such methods where one reduces the detailed mechanistic model of a complex biological network depending on the time scales of the reactions in the concerned system and as a result effectively cut short the number of molecular species and reactions to simulate stochastically [15,19]. In this context, one should keep in mind that well-separated timescales in a deterministic model do not guarantee a reduced QSSA model to be valid and there are instances where additional conditions needed to be fulfilled [20]. In last few decades, quasi-steady-state approximated models of different biological systems were proved to be quite successful to furnish all the deterministic dynamical features of the corresponding biological systems under different biological conditions and even in some cases the stochastic behavior as well [15,19,20]. There were evidences that some of these QSSA models were faithfully reproducing the intrinsic noise for simple biological systems [15,19,20]. In this context, we want to investigate what happens if we use the QSSA approach to quantify the intrinsic noise for relatively complex biological networks? Under what conditions the QSSA approach will quantify the intrinsic noise as accurately as the SSA performed using the mechanistic version of the model and when the result will differ significantly?

The organization of the paper is as follows. In the first section, we develop a mechanistic and the corresponding QSSA model for a simple positive feedback module. We employ Gillespie’s stochastic algorithm [16] to simulate the QSSA model as well as the mechanistic model to identify the conditions where both the models will give identical measure of intrinsic noise for the corresponding motif and where the results will start to differ from each other significantly. First, we compare the stochastic results obtained from the mechanistic and the corresponding QSSA models to investigate the role played by the mRNA and protein half-lives while keeping the protein and mRNA abundance levels fixed. In this section we changed the half-life values following two different approaches. In approach 1, we change the ratio of the mRNA and protein half-lives by keeping the mRNA half-life fixed [15] and in approach 2, we vary the absolute values of mRNA and protein half-lives by keeping the half-life ratio fixed. Next, we have done similar comparison between the two models as a function of number of molecules of protein (mRNA number of molecules remained fixed) and number of molecules of mRNA (protein number of molecules remained fixed) by following approach 1 and approach 2. We further perform similar studies with extended GRNs to show that the conclusions made from a simple positive feedback motif holds good even for relatively bigger networks as well. Finally, in the discussion section, we summarize our results and discuss the measures to be taken before using a QSSA model to faithfully quantify intrinsic noise for relatively complex biological networks.

### Creating mechanistic and QSSA models for a simple positive feedback module

**Fig 1:**
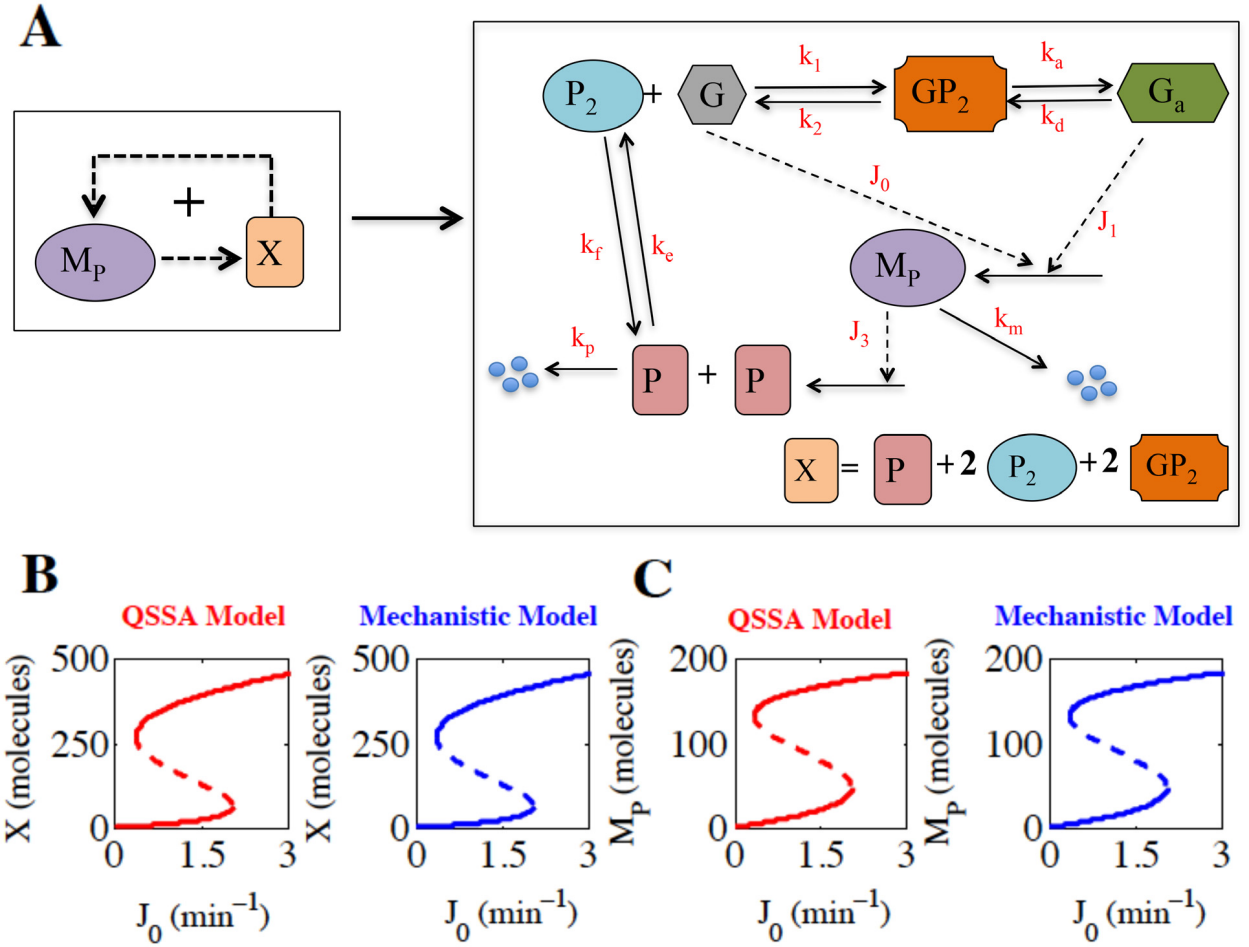
Positive feedback motif under consideration and the corresponding bifurcation diagrams. **(A)** QSSA positive feedback motif (left panel) and the detailed mechanistic scheme (right panel) are given where solid and dotted lines represent biochemical reactions and catalytic effects respectively. G and G_a_ denote inactive and active states of gene, M_p_ denotes mRNA, P denotes protein, P_2_ is the dimer of protein and X is the total protein. The protein (P) molecules form dimers P_2_. P_2_ binds to the promoter region of the inactive gene (G) and activates G to G_a_. *k*_*m*_ and *k*_*p*_ are the degradation rates of M_P_ and P respectively. **(B)** Bifurcation diagrams (left panel, QSSA model and right panel, mechanistic model) of total protein (X) are plotted as a function of *J*_*0*_. The solid lines represent stable steady states and dotted lines represent unstable steady states; *J*_*0*_ is the basal rate of mRNA (M_P_) synthesis and acts as the bifurcation parameter. In both the models deterministic mean of X (molecules) = 457.9 at *J*_*0*_ = 3 min^-1^. **(C)** Bifurcation diagrams (left panel, QSSA model and right panel, mechanistic model) of mRNA (M_P_) are plotted as a function of *J*_*0*_. In both the models deterministic mean of M_P_ (molecules) = 185.2 at *J*_*0*_ = 3 min^-1^.

We have constructed a toy model of a simple positive feedback motif [21–26] that is frequently observed in many biological regulations (Fig 1A, detailed network shown in the right panel) [25,26] in terms of mass action kinetics (mechanistic model, right panel, Table 1). We further made some appropriate steady state approximations on the variables except the total protein (X) and mRNA (M_P_) and obtained the corresponding QSSA model (left panel, Table 1) for the given GRN.

**Table 1:** Equations of the QSSA and Mechanistic models.

QSSA Model	Mechanistic Model
$\frac{{\text{dM}}_{\text{P}}}{\text{dt}}\;=\;\frac{J_{1}k_{a}\frac{\text{Y}}{k_{r}+\text{Y}}}{k_{d}+k_{a}\frac{\text{Y}}{k_{r}+\text{Y}}}+\frac{J_{0}}{1+\left(\frac{k_{d}+k_{a}}{k_{d}k_{r}}\right)\text{Y}}-k_{m}{\text{M}}_{\text{P}}$	$\frac{{\text{dGP}}_{2}}{\text{dt}}\;=\;k_{1}{\text{P}}_{2}\left({\text{G}}_{\text{t}}-{\text{G}}_{\text{a}}-{\text{GP}}_{2}\right)-k_{2}{\text{GP}}_{2}-k_{a}{\text{GP}}_{2}+k_{d}{\text{G}}_{\text{a}}$
$\frac{dX}{dt}\;=\;J_{3}M_{P}-k_{P}C$	$\frac{dG_{a}}{dt}\;=\;k_{a}{GP}_{2}-k_{d}G_{a}$
$\text{Y}\;=\;\left(\frac{\text{X}}{2}+\frac{1}{8K}-\sqrt{\left(\frac{\text{X}}{8K}+\frac{1}{64K^{2}}\right)}\right)$	$\frac{{\text{dM}}_{\text{P}}}{\text{dt}}\;=\;J_{1}{\text{G}}_{\text{a}}+J_{0}\left({\text{G}}_{\text{t}}-{\text{G}}_{\text{a}}-{\text{GP}}_{2}\right)-k_{m}{\text{M}}_{\text{P}}$
$\text{C}\;=\;\left(-\frac{1}{4K}+\sqrt{\left(\frac{\text{X}}{2K}+\frac{1}{16K^{2}}\right)}\right)$	$\frac{\text{dX}}{\text{dt}}\;=\;J_{3}{\text{M}}_{\text{P}}-k_{p}\text{P}-2k_{1}{\text{P}}_{2}\left({\text{G}}_{\text{t}}-{\text{G}}_{\text{a}}-{\text{GP}}_{2}\right)+2k_{2}{\text{GP}}_{2}$
$K\;=\;\frac{k_{e}}{k_{f}},\;k_{r}\;=\;\frac{k_{2}}{k_{1}}$	$\frac{{\text{dP}}_{2}}{\text{dt}}\;=\;-k_{1}{\text{P}}_{2}\left({\text{G}}_{\text{t}}-{\text{G}}_{\text{a}}-{\text{GP}}_{2}\right)+k_{2}{\text{GP}}_{2}+k_{e}{\text{P}}^{2}-k_{f}{\text{P}}_{2}$
	P = X– 2P_2_

The parameter values for the model concerned are given in Table 2. Here we have kept *k*_*m*_>>*J*_*3*_, as it was shown by Thomas et al. [20] that in order for the reduced QSSA model to be accurate one needs to fulfill this additional condition to be satisfied on top of the appropriate time scale separation. The details of the steady state approximations are provided in the S1 Text. The identical bifurcation diagrams shown in Fig 1B (left panel, QSSA model and right panel, mechanistic model) for the total protein (X) as a function of *J*_*0*_ (the basal rate of the M_P_ synthesis), signify that the two models provided in Table 1 are deterministically similar (the corresponding bifurcation diagrams of M_P_ as a function of *J*_*0*_ are given in Fig 1C). This clearly shows that both the deterministic models will lead to identical steady states deterministically under different values of *J*_*0*_ and sets the stage to investigate whether the QSSA model shown in Table 1 (left panel) can quantify the intrinsic noise as efficiently and accurately as the detailed mechanistic model (right panel, Table 1) or not.

**Table 2:** Parameters of the QSSA and Mechanistic models.

Parameter	Value
*J*_1_	43.838 min^-1^
*J*_3_	9.22E-04 min^-1^
*k*_1_	4.0E-01 molecule^-1^min^-1^
*k*_2_	81.31 min^-1^
*k_a_*	8.0 min^-1^
*k_d_*	5.0 min^-1^
*k_m_*	1.0E-01 min^-1^
*k_p_*	1.0E-03 min^-1^
*k_e_*	4.0E-01 molecule^-1^min^-1^
*k_f_*	81.31 min^-1^
G_t_	1 molecule

## Results

### Comparison of the stochastic distributions at different values of *J*_*0*_

**Fig 2:**
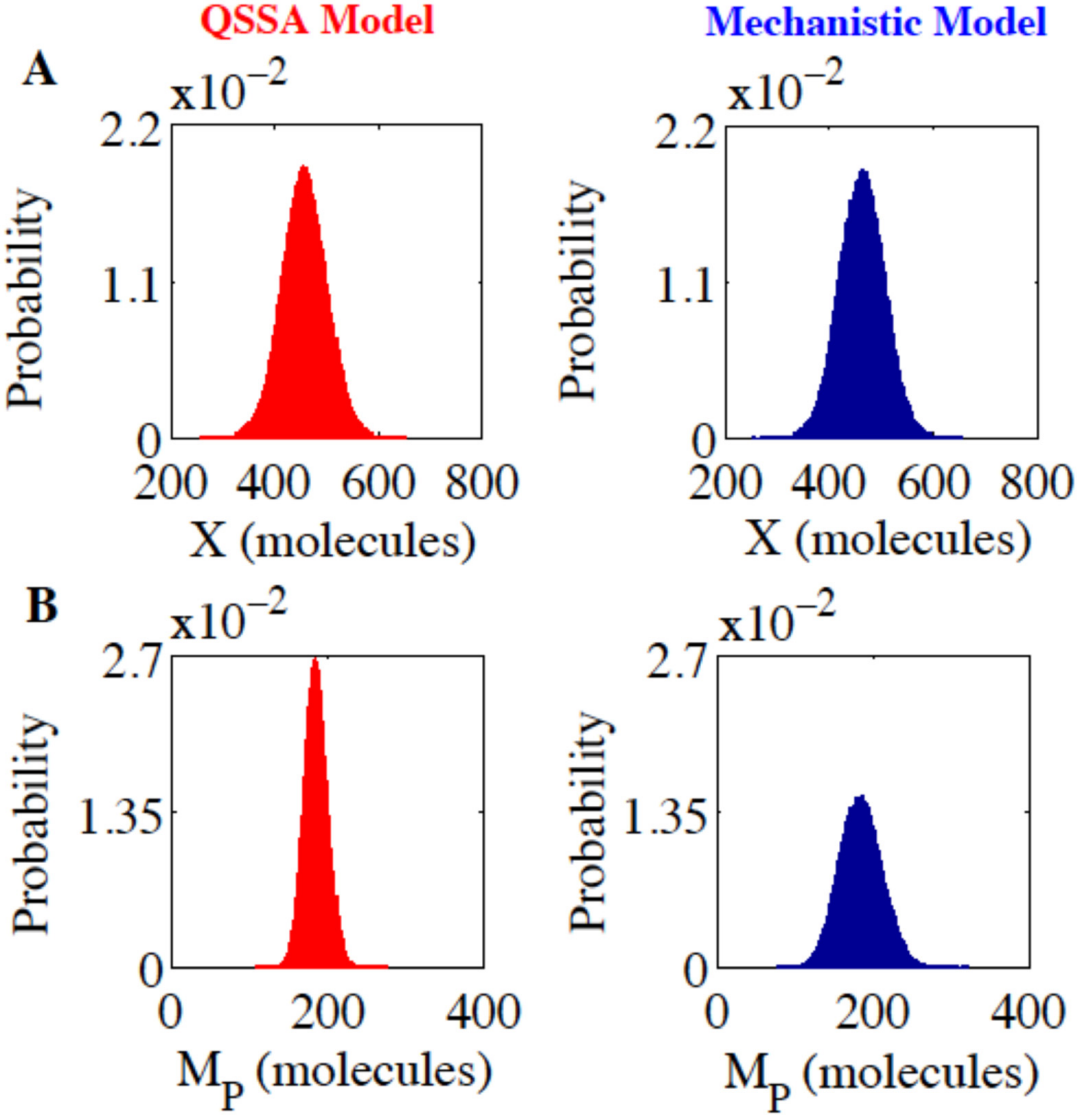
Stochastic simulation based on Gillespie Algorithm. **(A)** Steady state distributions of X (total protein) are plotted at *J*_*0*_ = 3 min^-1^ for the QSSA (left panel, mean = 457.2, standard deviation = 42.98, CV = 9.4%) and the mechanistic models (right panel, mean = 462.3, standard deviation = 43.54, CV = 9.42%). **(B)** Steady state distributions of M_P_ (mRNA) are plotted at *J*_*0*_ = 3 min^-1^ for the QSSA (left panel, mean = 185.2, standard deviation = 15.05, CV = 8.13%) and the mechanistic models (right panel, mean = 185.6, standard deviation = 27.26, CV = 14.68%). All the values (except CVs) are in number of molecules.

Once we created the deterministically similar models, we employed the Gillespie’s simulation algorithm [16] at *J*_*0*_ = 3 min^-1^ (deterministic means of X = 457.9 molecules and M_P_ = 185.2 molecules) to quantify the intrinsic noise from both the models (The reaction scheme followed for Gillespie simulation for both the models are provided in the S1 Text). We have performed the stochastic simulation at *J*_*0*_ = 3 min^-1^ where we do have only one stable steady state and measured the steady state distribution of the total protein (X) to compare the QSSA (left panel, Fig 2A) and mechanistic (right panel, Fig 2A) models (The corresponding time courses for the two situations are given in S1A Fig). The result looks extremely promising from the perspective of the QSSA model as the statistical quantities (mean = 457.2 and standard deviation = 42.98 in number of molecules, Coefficient of variation (CV) = 9.4%) obtained from the total protein distribution resembles quite well with the statistical values (mean = 462.3 and standard deviation = 43.54 in number of molecules, CV = 9.42%) obtained from the corresponding total protein distribution of the mechanistic model.

This evidently shows that under the chosen parameter regime the QSSA model does a good job to accurately quantify the molecular noise at the protein level in comparison to the mechanistic model. This kind of Gillespie runs is also performed with different sets of random number sequences (one of them shown in S1C Fig) and we got similar results. Additionally, we make sure that the parameter set used for the steady state approximation to obtain the QSSA model is a sensible choice. Since, if one performs Gillespie simulation with a wrong choice of parameters (for example, *k*_*a*_ = 8E-03 min^-1^ and *k*_*d*_ = 5E-03 min^-1^ instead of *k*_*a*_ = 8 min^-1^ and *k*_*d*_ = 5 min^-1^ as given in Table 2, Fig 2A), the stochastic results (S1 Table) for the mechanistic model (S2 Fig, right panel) will be totally different than that of the stochastic simulation result (S1 Table) of the corresponding QSSA model (S2 Fig, left panel). Stochastic results for the QSSA model are not at all affected by the inaccuracies made in the steady state approximations; where as mechanistic model can sense such changes quite adequately.

Although we got similar total protein distributions for the two models (Fig 2A), the corresponding mRNA (M_P_) steady state distributions that are given in Fig 2B (left panel, QSSA model and right panel, mechanistic model) appear to be quite different in nature (The corresponding time courses for the two situations are given in S1B Fig). We observe that the statistical quantities such as standard deviation and the CV at the mRNA level (mean = 185.2, standard deviation = 15.05, CV = 8.13%, for QSSA model and mean = 185.6, standard deviation = 27.26, CV = 14.68%, for mechanistic model) are quite different for the two models under the same parametric domain even though the average abundance level of M_P_ is same for both the cases. It clearly indicates that the protein (P) in the given biological network is not sensing the molecular noise present at the mRNA level adequately in case of the mechanistic model. As a consequence the intrinsic noise calculated in the form of CV of the total protein distribution for QSSA and mechanistic models seems to be in agreement. For other values of *J*_*0*_ (for example *J*_*0*_ = 1.5 min^-1^, S3 Fig) corresponding to the bi-stable domain sometimes we have found subtle differences (S3A Fig) and sometimes the results match quite well (S3B–S3D Fig) between the two models depending on whether we start the simulations from lower or upper steady states for a particular random number sequence. Since in the bi-stable domain it is hard to quantify and compare the noise statistics in a systematic manner, at this juncture we concentrate to understand why in case of the mechanistic model, the total protein level is not sensing the molecular noise at the mRNA level adequately at *J*_*0*_ = 3 min^-1^ for the current parameter set given in Table 2? We believe, this will eventually lead to the answer that under what conditions the QSSA model for the simple positive feedback motif will give similar result stochastically and will efficiently quantify the intrinsic noise like its detailed mechanistic version.

### Comparison of intrinsic noise as a function of the half-lives of the mRNA (M_P_) and total protein (X)

In literature [3,4,14,15], it is well known that the half-lives of the mRNA and protein play a crucial role to determine the noise at the protein level. Pedraza and Paulsson [14] had shown that for simple transcription and translational events the intrinsic noise at the protein level is given by,

$${\left({\text{CV}}_{\text{P}}\right)}^{2}\;=\;\frac{1}{\langle N_{\text{P}}\rangle }+\left(\frac{\tau_{\text{M}}}{\tau_{\text{M}}+\tau_{\text{P}}}\right)\frac{1}{\langle N_{\text{M}}\rangle \;}$$ (1)

where, CV_P_ is the coefficient of variation of the protein distribution, which not only depends on the average number of protein (<*N*_P_>) and mRNA (<*N*_M_>) molecules but it is also highly dependent on the half-lives *τ*_P_ and *τ*_M_ of the corresponding protein and mRNA molecules. Keeping these observations in mind, we wanted to verify whether the dependence on half-lives still exist in a gene regulatory network with feedback regulation or not and whether the stochastic calculation with QSSA model can capture such effects effectively or not like a detailed mechanistic model. At this point, we have taken two different approaches to vary the half-lives of mRNA and protein molecules to compare the two models.

#### Approach 1: Varying the ratio of the half-lives (K_C_) by keeping the absolute value of the mRNA half-life fixed

**Fig 3:**
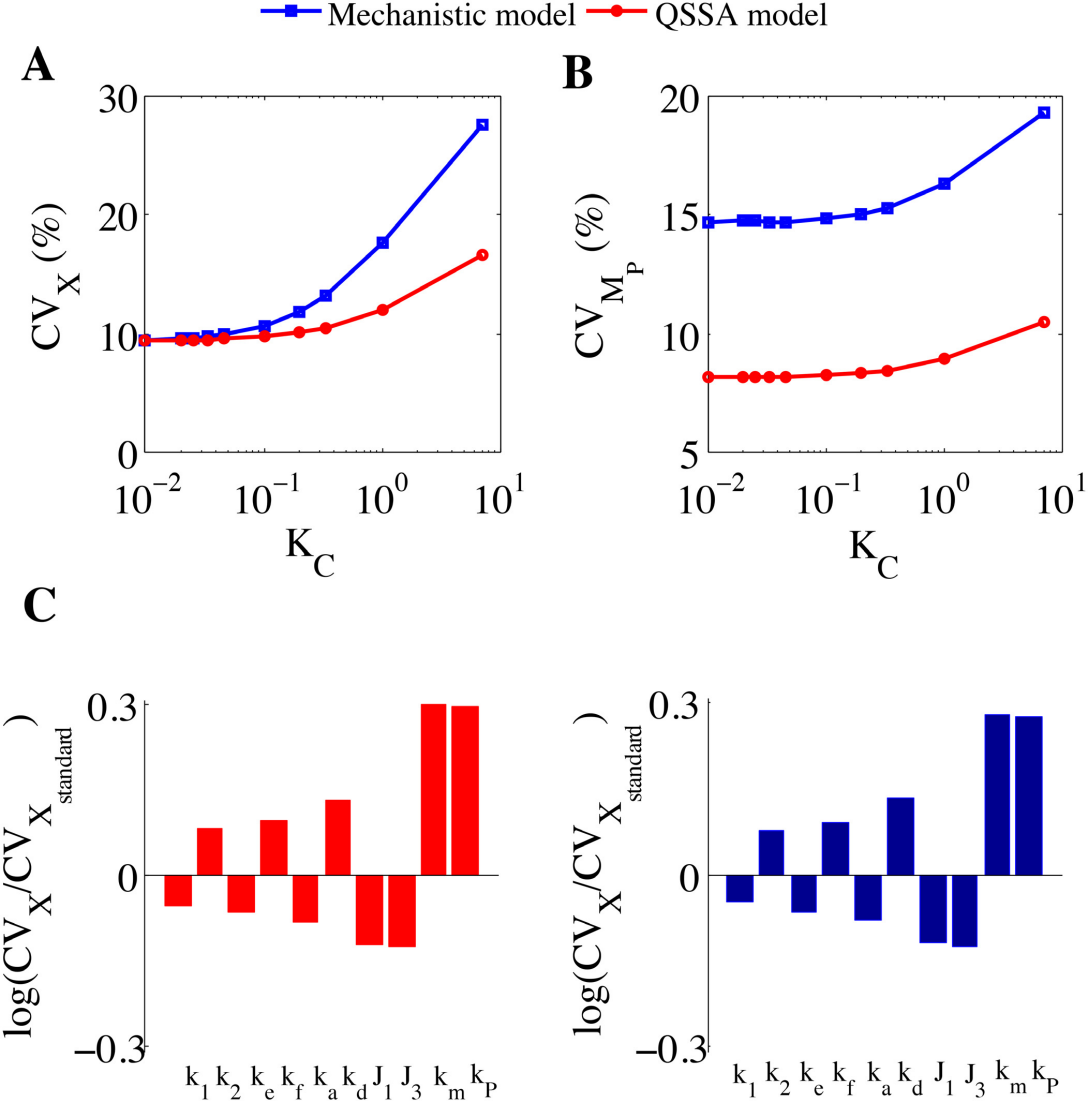
Plot of CV of protein and mRNA versus ratio of half-lives (K_C_) of M_P_ and P at *J*_*0*_ = 3 min^-1^ and the corresponding sensitivity analysis. **(A)** Coefficient of variation of total protein (X) abundance versus K_C_. **(B)** Coefficient of variation of M_P_ abundance versus K_C_. *k*_*m*_ = 1E-01 min^-1^ is considered in all the cases. When K_C_ = 0.01, then *k*_*p*_ = 1E-03 min^-1^, *J*_*3*_ = 9.22E-04 min^-1^. When K_C_ = 0.02, then *k*_*p*_ = 2E-03 min^-1^, *J*_*3*_ = 1.85E-03 min^-1^. When K_C_ = 0.025, then *k*_*p*_ = 2.5E-03 min^-1^, *J*_*3*_ = 2.31E-03 min^-1^. When K_C_ = 0.033, then *k*_*p*_ = 3.34E-03 min^-1^, *J*_*3*_ = 3.08E-03 min^-1^. When K_C_ = 0.046, then *k*_*p*_ = 4.67E-03 min^-1^, *J*_*3*_ = 4.3E-03 min^-1^. When K_C_ = 0.1, then *k*_*p*_ = 1E-02 min^-1^, *J*_*3*_ = 9.22E-03 min^-1^. When K_C_ = 0.2, then *k*_*p*_ = 2E-02 min^-1^, *J*_*3*_ = 1.85E-02 min^-1^. When K_C_ = 0.33, then *k*_*p*_ = 3.34E-02 min^-1^, *J*_*3*_ = 3.08E-02 min^-1^. When K_C_ = 1.0, then *k*_*p*_ = 1E-01 min^-1^, *J*_*3*_ = 9.22E-02 min^-1^. When K_C_ = 7.0, then *k*_*p*_ = 7E-01 min^-1^, *J*_*3*_ = 6.5E-01 min^-1^. All other parameters are same as Table 2. **(C)** Sensitivity analysis of the parameters involved at K_C_ = 1E-02 and *J*_*0*_ = 3 min^-1^. Sensitivity is measured on the basis of CV of stochastic mean of X as a function of rate constants involved in QSSA model and mechanistic model. Here CV_X_ refers to the coefficient of variation in stochastic mean of X resulting due to parameter variation. It is divided by the CV of our standard stochastic model (Fig 2A) using our model-parameter set (Table 2). All the parameters are increased individually at an amount of 10% of the model parameters (Table 2) keeping all other parameters constant.

We performed a systematic study where we have calculated the CV for the total protein and mRNA distributions as a function of different ratio (KC = τMτP) of mRNA and protein half-lives keeping the abundance levels of the protein (~ 458 molecules) and mRNA (~ 185 molecules) fixed (deterministically) for all the ratios of half-lives used (Fig 3 and S2 Table).

This approach is similar to some earlier study performed by Shahrezaei and Swain [15] for simple transcription and translational regulations of a protein (without any feedback regulation) where they have shown that if γ > 1 (where γ = 1KC according to our definition) then the analytical expression obtained by them for the noise at the protein level after QSSA on the mRNA dynamics, nicely fits the data for budding yeast experimental observations [13,27,28]. To obtain Fig 3A and 3B, we fixed the value of the mRNA half-life at *τ*_M_ = 7 min [29] (in Fig 2 we have used *τ*_M_ = 7 min, as *k*_*m*_ = 1E-01 min^-1^) and varied protein half-life *τ*_P_ (from 700 min to 1 min) [30] that eventually varies the ratio K_C_. From Fig 3A, it is quite evident that for K_C_<1, i.e. when the half-life of protein is much greater than the mRNA half-life, the stochastic calculation obtained from QSSA model resembles the stochastic result generated from mechanistic model if we compare the CV of total protein (X). CV_X_ in case of stochastic calculation from QSSA model only starts to deviate significantly from that of the mechanistic version when the protein half-life is taken 35 mins (K_C_ = 0.2) (Fig 3A and S2 Table). This indicates that in our case as the ratio K_C_ becomes greater than 0.1, the mechanistic model starts to sense the molecular fluctuations due to mRNA more efficiently and hence the stochastic quantifications done from the two models start to differ. As the ratio K_C_ is increased the CV_X_ is also rising in case of the stochastic calculations done from the QSSA model and it shows similar trend as that of the mechanistic model but the rise is not as steep as it is in case of the mechanistic model simulations (Fig 3A). This is expected as we can see in Fig 3B (S2 Table) that the stochastic simulations from QSSA model cannot capture the molecular noise at the mRNA level even for the lowest magnitude of the ratio K_C_ in comparison to the simulations performed with mechanistic model. We start to observe the effect of the differences in the mRNA CV levels found in Fig 3B at the CV_X_ for the two models (Fig 3A), only for higher values of K_C_. This shows that the stochastic runs with QSSA model for the considered network are good to understand the trend of the intrinsic noise variation for the total protein level but it appears from Fig 3A that they are not appropriate for accurate quantification of the intrinsic noise if the ratio of the half-lives of the corresponding mRNA and protein are such that we have K_C_>0.1 but for K_C_≤0.1 the stochastic results from QSSA model will be quite reliable. At this juncture we did perform sensitivity analysis [31,32] of the rate constants involved in the QSSA and mechanistic models by taking the corresponding associated CVs for the total protein (X) (Fig 3C) at *J*_*0*_ = 3 min^-1^. We found that for K_C_ = 0.01 situation, both the QSSA and mechanistic model parameters show quite identical sensitivities (Fig 3C) for all the rate constants related to the system, so no wonder that the stochastic calculation executed with the QSSA model gives similar results like the mechanistic model for K_C_ = 0.01 (Fig 3A). The sensitivity analysis performed with K_C_ = 0.2 (S4A Fig) and K_C_ = 1 (S4B Fig) start to differ for QSSA and mechanistic models, which kind of supports why the CVs of total protein quantified from the two models start to vary as we increase the value of K_C_ further in Fig 3A.

#### Approach 2: Varying the absolute values of the mRNA and protein half-lives keeping the ratio K_C_ fixed

Till now we have discussed if we change the ratio K_C_ (by keeping *τ*_M_ = 7 min and changing only *τ*_P_) how good a QSSA model performs in comparison to the mechanistic model to quantify the intrinsic noise. This kind of calculation makes sense for the case of budding yeast where for more than 80% of genes the value of K_C_<1 [15,27,28]. However, it is well-known that the absolute values of the half-lives (*τ*_M_ and *τ*_P_) for mRNA and protein are equally inportant to the level of gene expression noise [3,4,14].

**Fig 4:**
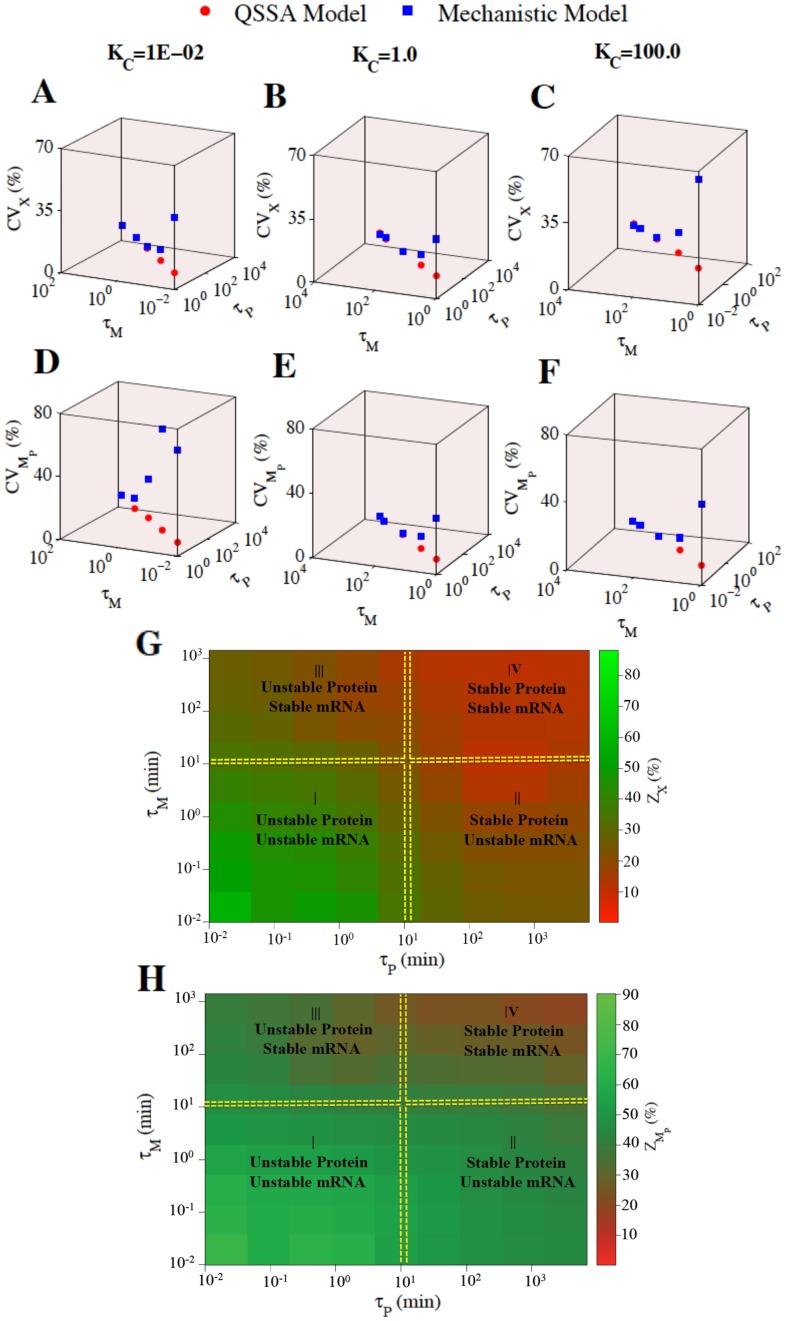
Impact of absolute values of τP and τM on CV of X (Total protein) and MP (mRNA) as well as on ZX and $Z_{M_{P}}$ (where Zi = % deviation of the measured intrinsic noise between the QSSA and the mechanistic models for i = X or MP). Plot of CV of X versus *τ*_P_ and *τ*_M_ for **(A)** K_C_ = 1E-02 **(B)** K_C_ = 1.0 **(C)** K_C_ = 100.0. Plot of CV of M_P_ versus *τ*_P_ and *τ*_M_ for **(D)** K_C_ = 1E-02 **(E)** K_C_ = 1.0 **(F)** K_C_ = 100.0. **(G)** Impact of absolute values of *τ*_P_ and *τ*_M_ on Z_X_. **(H)** Impact of absolute values of *τ*_P_ and *τ*_M_ on $Z_{M_{P}}$. *τ*_P_ and *τ*_M_ are the half-lives of P and M_P_ respectively and are given in minutes.

This implies that one can have fixed value of K_C_ for different values of *τ*_M_ and *τ*_P_, which can eventually change the burst size and burst frequencies and impact the overall intrinsic noise significantly. The important question is whether stochastic calculation from QSSA model can capture that feature accurately or not? To address this issue, we keep on changing the absolute values of the half-lives (*τ*_M_ and *τ*_P_) for mRNA and protein by keeping the ratio K_C_ fixed at 0.01 (Fig 4A and 4D), 1 (Fig 4B and 4E) and 100 (Fig 4C and 4F) (the average number of the total protein (~ 458 molecules) and mRNA (~ 185 molecules) are also kept fixed for all the cases deterministically, parameters are given in S2 Table). The way we varied the absolute values of the half-lives *τ*_M_ and *τ*_P_ (with K_C_ fixed at 0.01, 1, 100) span almost all the possibilities of half-life combinations that can exist in reality for mammalian cells found in recent experiment [30]. The stochastic simulation results obtained for both the models performed equally well to quantify intrinsic noise at the total protein (X) level for all the K_C_ values used in Fig 4A–4C for a certain range of absolute values of *τ*_M_ and *τ*_P_. For K_C_ = 0.01 (Fig 4A), the intirnsic noise quantified from QSSA model at the protein (X) level starts to differ from mechanistic model when *τ*_M_ = 0.1 min and *τ*_P_ = 10 min. Similarly for K_C_ = 1 (Fig 4B), the deviation starts at *τ*_M_ = 7 min and *τ*_P_ = 7 min and for K_C_ = 100 (Fig 4C), the deviation starts at *τ*_M_ = 10 min and *τ*_P_ = 0.1 min. Interestingly, we found that in Fig 4D–4F for certain combinations of absolute values of *τ*_M_ and *τ*_P_ (at different fixed values of K_C_), the stochastic QSSA model can also quantify the intrinsic noise at the mRNA (M_P_) level with reasonable accuracy in comparison to the mechanistic model. In all the cases (Fig 4A–4F), the CVs obtained from stochastic calculation of the QSSA model hardly changes for a fixed value of K_C_ when the absolute values of the *τ*_M_ and *τ*_P_ are changed whereas the CVs calculated from the mechanistic model starts to deviate from QSSA model as soon as the value of either *τ*_M_ or *τ*_P_ or both *τ*_M_ and *τ*_P_ are such that they can significantly change the burst frequency and burst size related to the intrinsic noise of the concerned network. This evidently shows an important fact that even for ratio K_C_>0.1 the QSSA model can quantify the intrinsic noise (for protein as well as for mRNA) as accurately as the mechanistic model and we can have situations where for ratio K_C_<0.1 the QSSA model can fail to quantify the intrinsic noise (even at the protein level) as precisely as the mechanistic model. This result is quite different than what has been shown by Shahrezaei and Swain [15] earlier as they did not concentrate on changing the absolute values of *τ*_P_ and *τ*_M_ while performing their analysis.

To understand this issue in more comprehensive manner, we further plotted the % deviation of the measured intrinsic noise (Z_i_) (Where Z_i_ = [(CV_i_ (mechanistic model)–CV_i_ (QSSA model)) / CV_i_ (mechanistic model)] x 100, i = X or M_P_) at the protein (X) (Fig 4G) and mRNA (M_P_) (Fig 4H) level in 2-dimentional heatmaps as a function of absolute values of *τ*_P_ and *τ*_M_. In Fig 4G and 4H, we have defined 4 different regions by considering the stabilities of the mRNA and the corresponding protein. We have made such classification by keeping the experimental data available for budding yeast [27,28] as well as the data for Mammalian cell [30] in mind. It is quite evident from Fig 4G that in region I (unstable mRNA/unstable protein) the stochastic results obtained from QSSA model deviates (>40–50% deviation) the most from the mechanistic model and in region IV (stable mRNA/stable protein), stochastic results generated from QSSA model are as good as (within 5–10% deviation) the mechanistic model with orders of magnitude reduction in computational time. The stochastic results obtained for the case of region II (stable protein/unstable mRNA) and region III (unstable protein/stable mRNA) by using the QSSA model are moderately satisfactory (10–30% deviation) in comparison to stochastic results from mechanistic model. Fig 4H also evidently shows that the intrinsic fluctuations at the mRNA level can only be captured faithfully by stochastic QSSA model for combinations of *τ*_P_ and *τ*_M_ values falling within region IV (within 10–20% deviation) and for a part of region (III) where the mRNA is highly stable. This clearly shows that even if there is enough separation of timescale between the mRNA and protein dynamics (as in case of region II and III), if either of them has a short absolute value of half-life then bursting frequency generated because of that will be hard to capture by stochastic simulation using QSSA model. On the contrary, The QSSA model will do a fine job to quantify the intrinsic noise for both mRNA and protein even if there is no separation of time scale between mRNA and protein dynamics (as in case of region IV) as long as the absolute values of *τ*_P_ and *τ*_M_ are such that the bursting frequencies generated due to them are negligible. As soon as the absolute values of *τ*_P_ and *τ*_M_ are quite small (region I) the effective increase in bursting frequency will change the intrinsic noise significantly and QSSA model will fail to capture that effect.

### Comparison of intrinsic noise as a function of number of protein molecules

**Fig 5:**
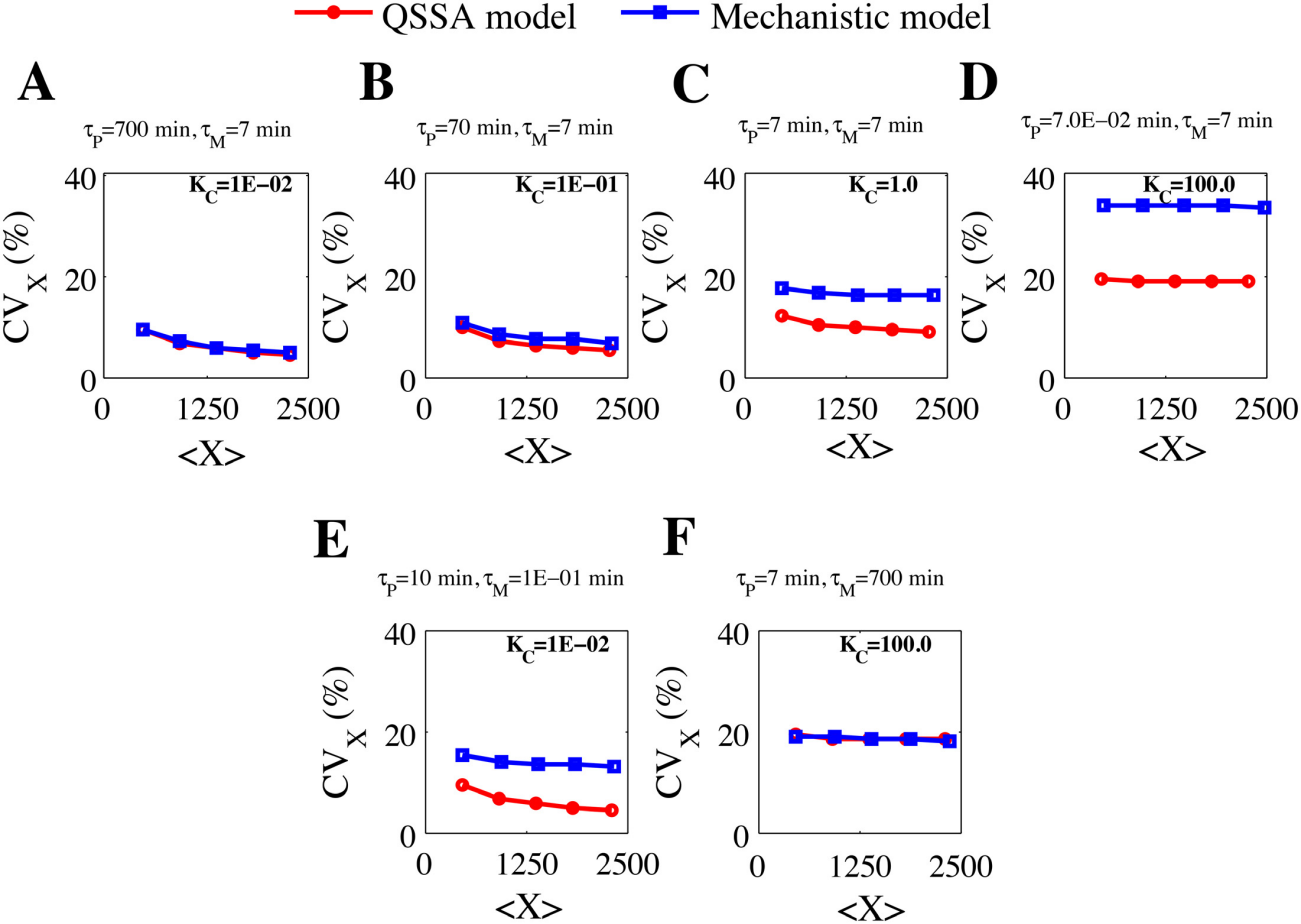
Plot of CV of total protein (X) versus total protein (X) abundance following approach 1 and approach 2 by keeping deterministic mean of M_P_ = 185.2 molecules for all the cases. **(A)** K_C_ = 1E-02, *τ*_P_ = 700 min (approach 1), **(B)** K_C_ = 1E-01, *τ*_P_ = 70 min (approach 1), **(C)** K_C_ = 1.0, *τ*_P_ = 7 min (approach 1), **(D)** K_C_ = 100.0, *τ*_P_ = 7.0E-02 min (approach 1). In all the cases we kept *τ*_M_ = 7 min. **(E)** K_C_ = 1E-02, *τ*_P_ = 10 min, *τ*_M_ = 1E-01 min (approach 2). **(F)** K_C_ = 100.0, *τ*_P_ = 7 min, *τ*_M_ = 700 min (approach 2).

In this section we wanted to understand how the QSSA model competes with the mechanistic model to capture the intrinsic noise as a function of number of protein molecule for a fixed number of mRNA molecules and fixed value of K_C_ (Fig 5A–5D, approach 1). We will further investigate what happens if we change the absolute values of the *τ*_P_ and *τ*_M_ by keeping the K_C_ fixed (Fig 5E and 5F, Approach 2).

The changes made in the parameter values (than given in the Table 2) to keep the deterministic mean of mRNA fixed ~ 185 molecules in all the cases for the Fig 5, are given in S3 Table. In Fig 5, we have shown how the CV of the total protein level will vary as a function of total protein abundance level keeping the deterministic mean of mRNA fixed at ~185 molecule at *J*_*0*_ = 3.0 min^-1^ for four different K_C_ values. For K_C_ = 0.01 (Fig 5A), the CV at the total protein level from the stochastic calculation obtained by using QSSA model seems to be identical as that obtained from mechanistic model as a function of total protein number. In both the cases the intrinsic noise at the total protein level decreases as the average number of protein molecule is increasing in a similar quantitative fashion. As the value of K_C_ is increased to 0.1 (Fig 5B), 1.0 (Fig 5C) and 100.0 (Fig 5D), the statistical results quantified from stochastic calculation of QSSA model started to differ from the stochastic results obtained from the mechanistic model quantitatively. The qualitative feature of the stochastic calculation from QSSA model still showed similar trend as that of the stochastic result of the mechanistic model. We have performed similar studies to compare the intrinsic noise at the total protein level as a function of average values of total protein by fixing the number of mRNA molecules at lower and higher levels (S5 Fig, related changes in the parameter values given in S4 Table) and it strengthens the conclusions made from Fig 5. From this study it is evident that if the half-lives ratio of mRNA and protein remains as K_C_≤0.1, then the stochastic results using QSSA model will definitely quantify the intrinsic noise at the total protein level as accurately as stochastic calculation done with the mechanistic model even if the protein number starts to vary significantly in the corresponding biological network. It does not mean that the intrinsic noise calculated from QSSA model for K_C_ = 100.0 (i.e., K_C_>0.1case) will always be inaccurate in comparison to mechanistic model and for K_C_<0.1 will always match. As we observed in case of Fig 4G, here too, if we measure the intrinsic noise as a function of total protein number using QSSA and mechanistic models by changing the absolute values of the half-lives *τ*_P_ and *τ*_M_ keeping the K_C_ fixed, we found that even for K_C_<0.1 (Fig 5E, K_C_ = 0.01, *τ*_M_ = 0.1 min and *τ*_P_ = 10 min) the results can differ significantly and for K_C_>0.1(Fig 5F, K_C_ = 100.0, *τ*_M_ = 700 min and *τ*_P_ = 7 min) the results can match quite well. This clearly demonstrates the fact that the absolute values of the protein and mRNA half-lives are the important dictating factors to decide whether the QSSA model is good enough to capture the intrinsic noise for a gene regulatory network in comparison to the corresponding mechanistic model or not, number of molecules of total protein hardly matters.

### Comparison of intrinsic noise as a function of number of mRNA molecules

In most of the biological systems, the abundance level of mRNAs is found to be much less than the corresponding protein molecules [1,3,12,30]. Keeping this in mind, next we wanted to investigate, how far the stochastic calculation from QSSA model can capture the effect due to the change in the mRNA numbers in comparison to the stochastic results obtained from mechanistic model at a fixed number of protein molecules and for different values of *τ*_P_ and *τ*_M_ (following both approach 1 and 2).

**Fig 6:**
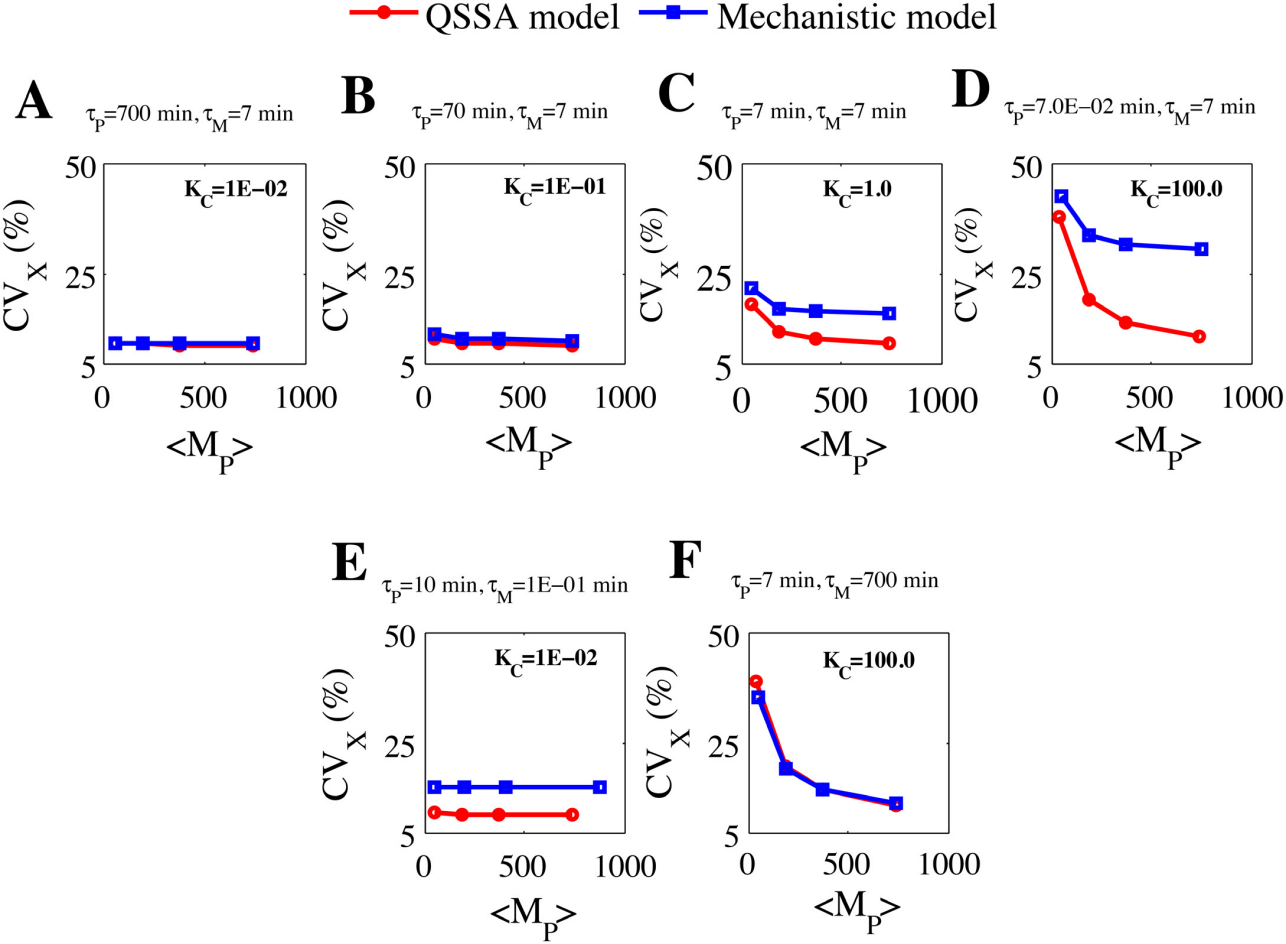
Plot of CV of total protein (X) versus mRNA (M_P_) abundance following approach 1 and approach 2 by keeping deterministic mean of X = 457.9 molecules for all the cases. **(A)** K_C_ = 1E-02, *τ*_P_ = 700 min (approach 1), **(B)** K_C_ = 1E-01, *τ*_P_ = 70 min (approach 1), **(C)** K_C_ = 1.0, *τ*_P_ = 7 min (approach 1), **(D)** K_C_ = 100.0, *τ*_P_ = 7.0E-02 min (approach 1). In all the cases we kept *τ*_M_ = 7 min. **(E)** K_C_ = 1E-02, *τ*_P_ = 10 min, *τ*_M_ = 1E-01 min (approach 2). **(F)** K_C_ = 100.0, *τ*_P_ = 7 min, *τ*_M_ = 700 min (approach 2).

Following approach 1, we fixed the values of K_C_ and kept the total protein level fixed at ~ 458 molecules in all the cases for the Fig 6A–6D (parameter values are given in the S5 Table). From Fig 6A it is evident that the CV calculated for the total protein level are comparable between the stochastic calculation done with QSSA and mechanistic models as a function of mRNA population when K_C_ (0.01) is small. As the K_C_ value is increased systematically by keeping the mean value of total protein fixed at ~ 458 molecules, the differences between the CV values at the total protein level (as a function of mRNA abundance) increased between the stochastic calculations performed with the QSSA and mechanistic models (Fig 6B with K_C_ = 0.1, Fig 6C with K_C_ = 1.0 and Fig 6D with K_C_ = 100.0). The qualitative nature of the variations in CV values remain identical in both the cases but quantitatively they again start to differ as a function of average number of mRNA molecules for K_C_>0.1. In this case too, it is quite clear from Fig 6E and 6F that if we change the absolute values of *τ*_P_ and *τ*_M_ (by keeping K_C_ fixed), the stochastic results calculated from QSSA model can differ for K_C_ = 0.01 (Fig 6E, *τ*_M_ = 0.1 min and *τ*_P_ = 10 min) and can be similar for K_C_ = 100.0 (Fig 6F, *τ*_M_ = 700 min and *τ*_P_ = 7 min) as a function of mRNA number.

This clearly shows that before using any QSSA model for quantifying the molecular noise faithfully, one must carefully check the absolute values of the half-lives of the proteins and mRNAs involved in that network and the number of molecules of mRNA and protein will be less influencing factors for this purpose.

### Considering the effect of additional feedback loops in the simple positive feedback module

**Fig 7:**
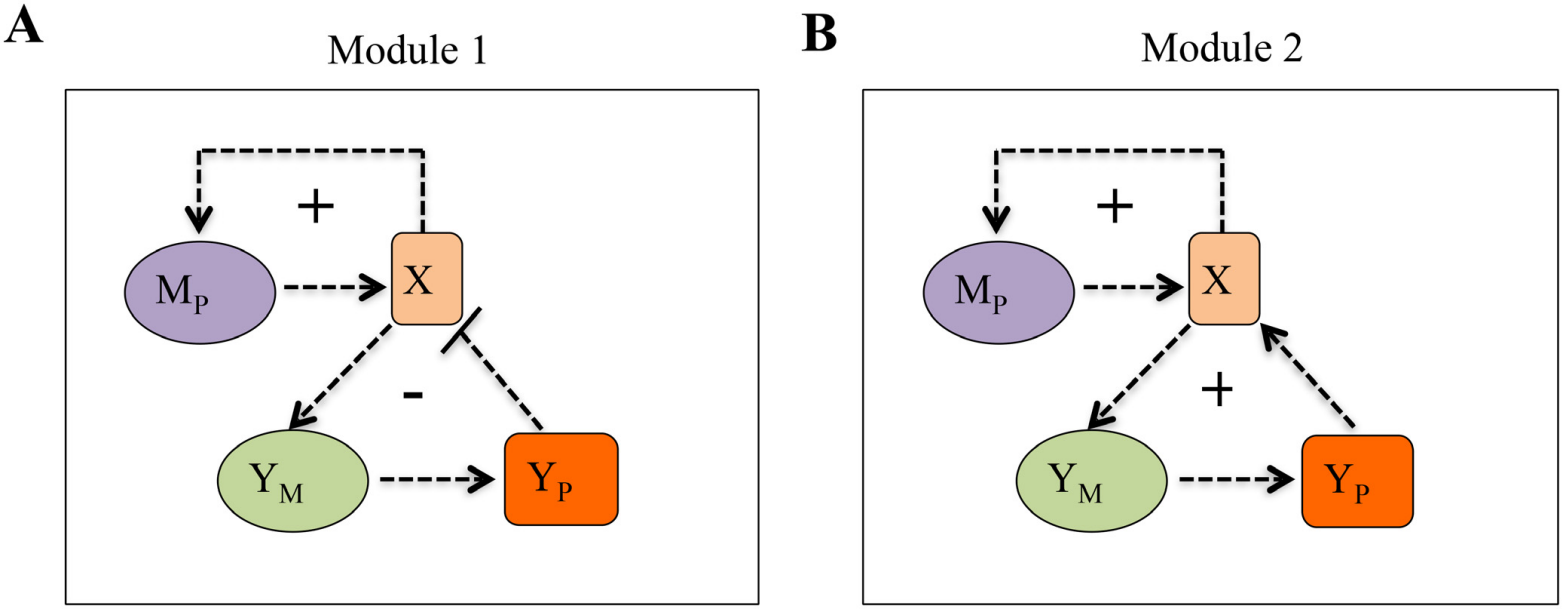
Various gene regulatory networks under consideration. **(A)** Positive feedback with additional negative feedback motif, where X (total protein) activates the synthesis of another protein Y_P_ which negatively regulates X population level. **(B)** Double positive feedback motifs, where X (total protein) activates the synthesis of another protein Y_P_ which positively regulates the synthesis of X. In all the cases X autoregulates its own synthesis positively. Here M_P_ denotes mRNA of P, Y_P_ denotes another protein and Y_M_ is the mRNA of protein Y_P_. Detailed mechanistic models are given in (S6 Fig).

To generalize the observations made in the previous sections, we have further systematically constructed mechanistic and QSSA models of two more modules (Fig 7). We have assumed that the protein (X) involved in the positive feedback module can further activate a protein (Y_P_) which inturn can deactivate (Fig 7A) or activate (Fig 7B) the production of protein (X) by giving a feedback at the protein (X) level (Fig 7A, negative feedback and Fig 7B, positive feedback).

We often encounter this kind of biological networks in different biological systems [33–35] and it is imperative to understand the reliability of the stochastic calculations using QSSA models to accurately evaluate the intrinsic noise regulations for such systems. The detailed version of the modules shown in Fig 7 are provided in S6 Fig and the corresponding QSSA models and the mechanisitc models along with the parameter values are given in the supplementary information (S6 and S8 Tables). We are interested in understanding how the molecular noise at the total protein (X) and mRNA (M_P_) levels will be influenced by the absolute values of $\tau_{Y_{M}}$ (half-life of Y_M_), $\tau_{Y_{P}}$ (half-life of Y_P_) and their ratio K_S_ (where(KS = τYMτYP)) by following the two approaches discussed in the earlier sections. Further, we would like to investigate how far the stochastic simulations based on the QSSA models constructed for the two modules shown in Fig 7 can reproduce the stochastic simulation results obtained from the corresponding mechanistic models under these situations.

#### Comparison of intrinsic noise as a function of the half-lives of proteins and mRNAs (for Module 1)

**Fig 8:**
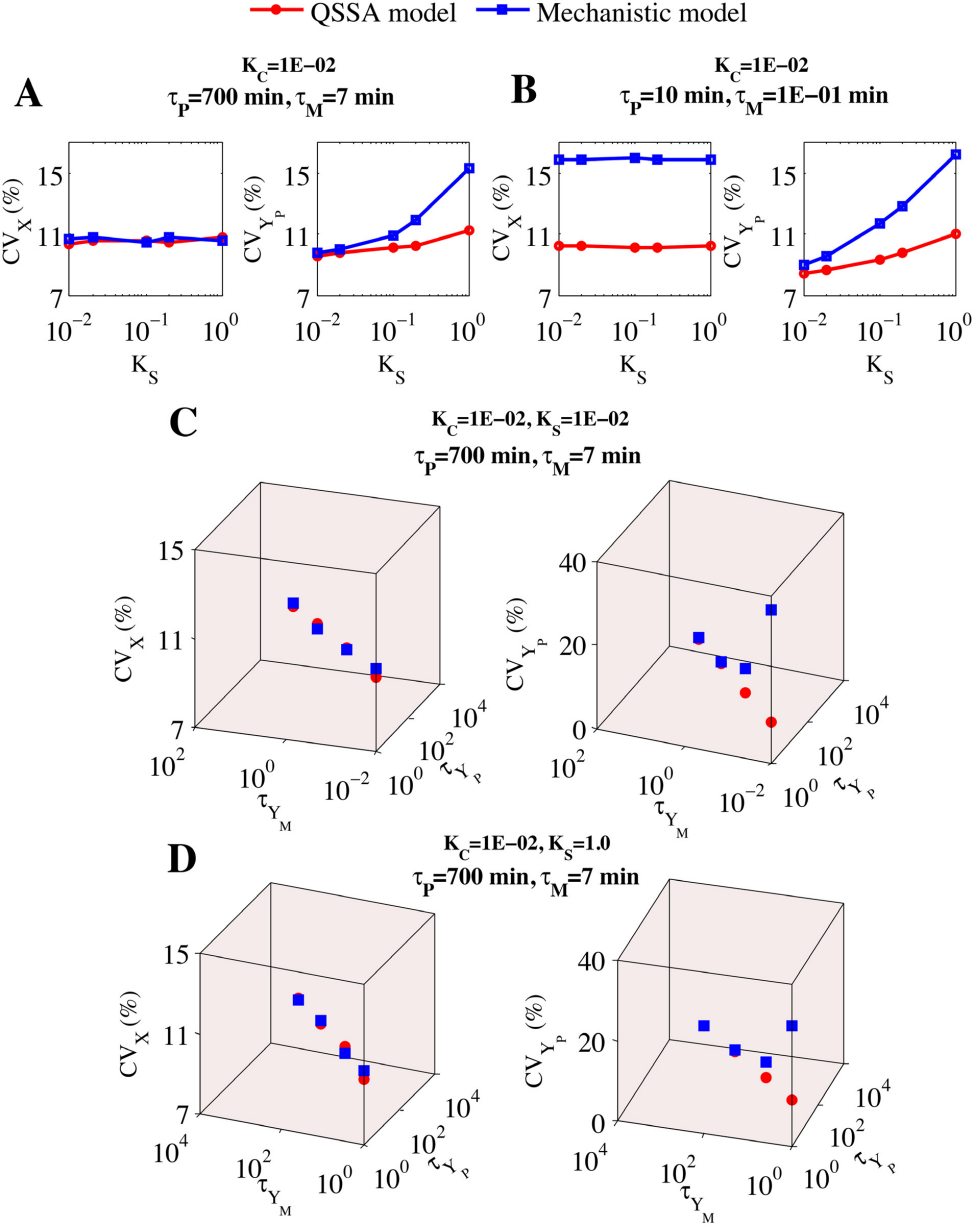
Plot of CV of the total protein (X) and Y_P_ versus ratio of half-lives (K_S_) of Y_M_ and Y_P_ as well as the absolute values of the half-lives of Y_M_ and Y_P_ at K_C_ = 1E-02 in the module 1 with additional negative feedback on X. **(A)** *τ*_P_ = 700 min, *τ*_M_ = 7 min and $\tau_{Y_{M}}$ = 7 min **(B)** *τ*_P_ = 10 min, *τ*_M_ = 1E-01 min and $\tau_{Y_{M}}$ = 7 min. **(C)** K_S_ = 1E-02, *τ*_p_ = 700 min and *τ*_M_ = 7 min. **(D)** K_S_ = 1.0, *τ*_P_ = 700 min and *τ*_M_ = 7 min. Values of $\tau_{Y_{M}}$ and $\tau_{Y_{P}}$ are given in minutes.

With the small positive feedback module studied earlier, we have observed that the intrinsic noise at the total protein level (X) as well as at the mRNA (M_P_) level can be evaluated nicely by the QSSA model in comparison to the mechanistic model under certain restricted domain of absolute values of the corresponding protein and mRNA half-lives (approach 2) involved in the network. To further investigate in this direction, we took a step forward and considered the existence of an additional negative feedback loop of Y_P_ on X in presence of positive feedback of X on its own transcription (for example, in mammalian cell cycle E2F autocatalyzes its own transcription and also activates CycA which together with Cdk2 activates the degradation process of E2F [33]). Before doing the stochastic simulations, we first did the bifurcation analysis (S7A and S7B Fig (left panel QSSA model and right panel mechanistic model, parameters are given in S6 Table)) of the corresponding deterministic models to show that both the models are deterministically similar even when the additional negative feedback loop at the total protein (X) level is operative in the system. We have calculated the molecular noise (following approach 1) at the total protein (X) level by using the stochastic version of both the models (S1 Text) given in S7 Table for module 1 (Fig 7A) as a function of K_S_ at two different fixed values of K_C_ = 0.01(*τ*_M_ = 7 min and *τ*_P_ = 700 min, Fig 8A) and K_C_ = 1.0 (*τ*_M_ = 7 min and *τ*_p_ = 7 min, S8A Fig).

In Fig 8A, the CV for the total protein (X) calculated from QSSA model resembles the mechanistic model calculation but the result for the CV of Y_P_ protein differs as we go on changing the K_S_ (following approach 1, $\tau_{Y_{M}}$ fixed at 7 min) at a fixed value of the ratio K_C_ (parameters used to keep the protein and mRNA numbers fixed are given in S7 Table). We have performed the similar comparison for K_C_ = 1 (*τ*_M_ = 7 min and *τ*_P_ = 7 min) as well and found that for this value of K_C_ (S8A Fig), QSSA model can not quantify the intrinsic noise precisely for both X and Y_P_. This result is in agreement with what we have discussed earlier for the simple positive feedback motif and consistent with the findings of Shahrezaei and Swain [15].

At this point, we changed the absolute values of the half-lives (approach 2) of the protein (X) and mRNA (M_P_) by keeping the ratio K_C_ same in Fig 8B (*τ*_M_ = 0.1 min and *τ*_P_ = 10 min for K_C_ = 0.01) as well as in S8B Fig (*τ*_M_ = 700 min and *τ*_P_ = 700 min for K_C_ = 1) and again performed same comparison between QSSA and mechanistic models. We can clearly see that now the stochastic results from QSSA model fails to capture the intrinsic noise accurately for both X and Y_P_ protein in comparison to mechanistic model in Fig 8B. On the contrary, S8B Fig shows that intrinsic noise at the level of X are quite comparable even for K_C_ = 1 but in case of Y_P_ there is still disagreement. The reason behind this is quite clear from Fig 4G. In case of Fig 8A, the values of the *τ*_M_ (7 min) and *τ*_P_ (700 min) used fall in the top part of the region (II) where stochastic result from QSSA model are reasonably in good agreement with mechanistic model calculation whereas the values of the *τ*_M_ (0.1 min) and *τ*_P_ (10 min) used for Fig 8B fall in the region (I) where the disagreement between QSSA and mechanistic models is evident. In case of S8A Fig, the values used for *τ*_M_ and *τ*_P_ are both 7 mins respectively, which corresponds to region (I) and consequently there was disagreement and as soon as the values of *τ*_M_ and *τ*_P_ are both changed to 700 mins in S8B Fig, (coresponding to region IV) there was agreement between the stochastic results performed with QSSA and mechanistic models.

Further we wanted to investigate if we change the absolute values of the half-lives of the protein Y_P_ and mRNA Y_M_ at fixed values of K_S_ and K_C_, how stochastic calculation with QSSA model performs in comparison to mechanistic model. To do this we considered two different cases K_S_ = 0.01, K_C_ = 0.01 (Fig 8C) and K_S_ = 1, K_C_ = 0.01 (Fig 8D). Fig 8C (left panel) and Fig 8D (left panel), vividly show that whatever may be the absolute values of $\tau_{Y_{M}}$ and $\tau_{Y_{P}}$ (for K_S_ either fixed at 0.01 or at 1), the intrinsic noise at the level of protein X, can always be quantitatively reproduced by the QSSA model in comparison to mechanistic model even if the two pairs of absolute values of $\tau_{Y_{M}}$ and $\tau_{Y_{P}}$ fall in region (I) defined in Fig 4G. Under the same situation the QSSA model fails to quantify the intrinsic noise accurately at the level of protein Y_P_ (Fig 8C and 8D, right panel) whenever the pair of absolute values of $\tau_{Y_{M}}$ and $\tau_{Y_{P}}$ falls in region (I) corresponding to Fig 4G. Fig 8C (right panel) and Fig 8D (right panel) also clearly show that the QSSA model will quantify the intrinsic noise appropriately at the level of protein Y_P_ in comparison to mechanistic model if the absolute values of $\tau_{Y_{M}}$ and $\tau_{Y_{P}}$ fall in region (II) and (IV) defined in Fig 4G. To show that the stochastic QSSA model can even capture the mRNA fluctuations for module 1, we performed the comparison between stochastic QSSA and SSA calculation from mechanistic model for K_S_ = 1 and K_C_ = 1 (S8C Fig) by keeping the absolute values of *τ*_P_ = 700 min and *τ*_M_ = 700 min (to be in the region (IV) of Fig 4H). It is evident that the intrinsic fluctuations for the mRNA (M_P_) (S8C Fig, top right panel) can be captured beautifully by stochastic QSSA model for all the combinations of absolute values of $\tau_{Y_{M}}$ and $\tau_{Y_{P}}$ in different domains. Moreover, The stochastic QSSA model can satisfactorily quantify the intrinsic noise for mRNA (Y_M_) (S8C Fig, bottom right panel) if the combinations of half-life values ($\tau_{Y_{M}}$ and $\tau_{Y_{P}}$) correspond to region (IV) as mentioned in Fig 4H and the quantification starts to differ as soon as the combination of half-life values tend to fall in region (I) or (II).

We have performed similar studies with the Module 2 (with an additional positive feedback motif) and the detail results are provided in S2 Text and in the supplementary figures (S9–S11 Figs, S9 Table) referred there on. The stochastic simulation studies performed with Module 1 and Module 2, reiterate the fact that absolute values of the protein and mRNA half-lives will be the major factor to decide whether the stochastic simulation performed by a QSSA model can quantify the intrinsic noise as efficiently as mechanistic model or not.

## Discussion

In important biological processes such as cell cycle regulation [6,9,10], maintainance of stemness [36,37], differentiation regulation [36,37] etc., intrinsic noise has been shown to play crucial role to determine the ultimate cell fate. Not only that, intrinsic noise can even drive a system to a completely different dynamical regime which is not permitted if we analyze the corresponding deterministic model under same conditions [38,39]. One of the standard practice to tackle intrinsic noise theoretically is to construct a proper mechanistic model in terms of mass action kinetic terms for the corresponding gene regulatory network under consideration and employ the Gillespie stochastic simulation algorithm (SSA) [16] to accurately quantify the molecular noise present in the system. Unfortunately, in most of the cases the Gillespie simulation for a detailed mechanistic model is highly expensive in terms of computational time. In literature, efforts had been made [15,17–19] to make appropriate approximations to simulate only the slow reactions involved in a large biological network by SSA. Among them the QSSA method found to be quite successful in reducing the computational time for SSA without losing much the accuracy of the result for simple biological systems [15,19]. In this regard the pertinent question that we raised is that whether it is appropriate to use these QSSA models to quantify the intrinsic noise for gene regulatory networks with feedback motifs? How much reliable they are to quantify the molecular noise in comparison to the detailed mechanistic models? This is an important question because if the QSSA models do a decent job to quantify the intrinsic noise in comparison to mechanistic models, then computationally SSA calculations will become much faster and easy to execute for larger networks.

By following approach 1, We have demonstrated that for the simple positive feedback motif under consideration, the stochastic calculations of QSSA models can quantify the intrinsic noise for a particular protein in a network with reasonable accuracy as a function of the ratio (K_C_) of the half-lives of mRNA and protein, provided both the protein and mRNA are reasonably stable (which means less bursting frequency) and under the condition K_C_<0.1. We observed that after a certain specific value of the ratio K_C_ (K_C_>0.1), the stochastic results obtained from the QSSA model simulation for the protein (X) started to differ from the stochastic results of the mechanistic model simulation (Fig 3) which is quite in accordance with the observations made by Shahrezaei and Swain [15]. Although our approach 1 makes sense in the context of budding yeast where for more than 80% genes the protein half-life [13] is greater than the corresponding mRNA half-life [27,28], we have to keep in mind that even if the lifetimes of mRNA and protein are well separated in time scale (for the values K_C_<0.1) it might not guarantee that the reduced QSSA model will always be able to capture the intrinsic noise efficiently. We have further shown by implementing approach 2 that we can have disagreement for K_C_<0.1 and agreement for K_C_>0.1 between the two models and these things highly depend on the absolute values of the mRNA and protein half-lives. This result is quite unique and Shahrezaei and Swain [15] did not perform such analysis.

In this context, we have convincingly demonstrated using 2D heat maps (Fig 4G and 4H) that absolute values of the mRNA and protein half-lives are the crucial determining factors when one tries to compare the quality of the intrinsic noise quantified from a stochastic QSSA model in comparison to SSA performed using mechanistic model. We can have perfect agreement between the two calculations both at the protein and mRNA levels if the absolute values of the half-lives of the correposnding protein and the mRNA are located within region (IV) (as defined in Fig 4G or 4H). There will be a significant level of disagreement between the stochastic calculations performed from QSSA and mechanistic models if the absolute values of the half-lives correspond to region (I) i.e., when both the protein and mRNA are highly unstable causing a high degree of bursting frequency, which stochastic calculation performed from QSSA model will not be able to capture efficiently. As the half-lives of the proteins and mRNAs related to the gene regulatory network start to approach region (II) or (III), the success of the stochastic calculations using a QSSA model in comparison to mechanistic model will vary from case to case as shown in Fig 4G and 4H for both protein and mRNA respectively. This is a very important result in the context of recent experiments performed with mammalian cell [30] where they have shown that half-life combinations of protein and mRNA rarely turns out to be in the region (I) (as defined in Fig 4G) but most of the time the half-life combinations will fall in region (II), (III) and specially in region (IV) and beyond. This means that more often and not we can employ QSSA simplification for the corresponding mechanistic models of any gene regulatory network related to mammalian cell and have a good idea about the intrinsic noise if we take care of the additonal requirements as suggested by Thomas et al. [20].

We have further shown that the abundance level of protein (Fig 5) or mRNA (Fig 6) hardly influences the difference that we observe between the stochastic results obtained from QSSA and mechanistic models. To generalize our observations, we have further considered two different modules [33,34] (Fig 7) with additional negative and positive feedback loops and executed similar kind of studies as performed in case of simple positive feedback module shown in Fig 1A. These studies performed with module 1 and module 2 further corroborate the fact that absolute values of the half-lives of the proteins and mRNAs related to the gene regulatory network critically control the success of QSSA model in comparison to mechanistic model in the context of accuracy level achieved for intrinsic noise quantification. If the absolute values of all the half-lives (related to all the proteins and mRNAs involved in the extended GRNs) are such that they correspond to the region (IV) defined in Fig 4G and 4H, then we can definitely use the QSSA model to faithfully calculate the intrinsic noise for both protein and mRNA for a gene regulatory network. If they fall in region (II) or (III) then depending on other additional conditions the QSSA model might quantify the intrinsic noise with reasonable accuracy in comparison to its mechanistic counterpart but it will definitely fail to do so when the half-life values correspond to region (I).

In conclusion, our analysis with few frequently observed GRNs suggests that care must be taken before using any QSSA model for stochastic calculations although they are computationally less expensive. We must have an idea about the experimental half-lives of the proteins and mRNAs involved in the corresponding biological network before using a QSSA model to reliably quantify intrinsic noise for a GRN. We strongly believe that our finding about the importance of the absolute values of the half-lives while considering the efficiency of the stochastic QSSA model in comparison to SSA performed with mechanistic model is quite generic and will be applicable for even bigger GRNs. We hope that this work of us will shine some light to systematically use QSSA method for accurate measurement of intrinsic noise for large networks.

## Materials and Methods

### Deterministic simulations

The complete gene auto-regulatory network (Fig 1A) was expressed in terms of 5 ordinary differential equations in case of mechanistic model and 2 ordinary differential equations in case of QSSA model (Table 1). For simplicity we neglected the contribution of GP_2_ to total protein. As G+G_a_+GP_2_ = 1, so GP_2_ level was very low. Thus we can safely neglect the contribution of GP_2_ in total protein. The deterministic models were encoded as .ode files and deterministic simulations were done by the freely available software XPP-AUT. The bifurcation diagrams were drawn using AUTO facility available in XPP-AUT. The bifurcation diagrams shown in Fig 1 and S5 Fig were drawn in MATLAB using the data points generated by XPP-AUT.

In a similar fashion each of the other modules (Fig 7) was expressed in terms of 9 ordinary differential equations in case of mechanistic models and 4 ordinary differential equations in case of QSSA models (S6 and S8 Tables). Based on these ordinary differential equations we have constructed the .ode files. For simplicity contribution of GP_2_ and GP_2s_ to total protein population were neglected in all the cases. The bifurcation diagrams shown in S7 and S9 Figs were generated using the procedure discussed above.

### Stochastic simulations

We employed stochastic simulation based on Gillespie’s algorithm [16]. In the traditional deterministic analysis reaction constants are considered as reaction rates but in stochastic analysis the reaction constants are considered as ‘probabilities per unit time’ [16]. The mechanistic model corresponding to Fig 1 consisted of 11 reactions and the QSSA model consisted of 5 reactions. The mechanistic models of the modules mentioned in Fig 7 were expressed in terms of 21 reactions and the corresponding QSSA models were expressed in terms of 11 reactions in each case. Corresponding reactions and propensities of the reactions were given in S1 Text. We have stochastically simulated the models using different random number sequences. The plots shown in the figures were drawn in MATLAB using the data points generated by stochastic simulation.

## Supporting Information

S1 Fig Stochastic simulation based on Gillespie Algorithm. **(A)** In the left and right panels, we plot variation in population of X (total protein) with time in the QSSA model and mechanistic model respectively at *J*_*0*_ = 3 min^-1^. **(B)** In the left and right panels, we plot variation of M_P_ with time in the QSSA model and mechanistic model respectively at *J*_*0*_ = 3 min^-1^. **(C)** Stochastic simulation based on Gillespie Algorithm using another random number sequence. In the left and right panels, we plot steady state distribution of X (total protein) in the QSSA model at *J*_*0*_ = 3 min^-1^, mean = 457.3, standard deviation = 42.69, CV = 9.34% and steady state distribution of X (total protein) in the mechanistic model, mean = 461.4, standard deviation = 43.46, CV = 9.42% respectively. All the values (except CVs) are in number of molecules.
(TIFF)

S2 Fig Steady state analysis of QSSA and mechanistic models. In the left panel, we plot steady state distribution of X (total protein) in the QSSA model at *J*_*0*_ = 3 min^-1^, when *k*_*a*_ and *k*_*d*_ are 8E-03 min^-1^ and 5E-03 min^-1^, mean = 457.2, standard deviation = 42.98, CV = 9.4%. In the right panel, we plot steady state distribution of X (total protein) in the mechanistic model at *J*_*0*_ = 3 min^-1^, when *k*_*a*_ and *k*_*d*_ are 8E-03 min^-1^ and 5E-03 min^-1^, mean = 523.7, standard deviation = 197.6, CV = 37.7%. All the values (except CVs) are in number of molecules.
(TIFF)

S3 Fig Steady state distribution of X at *J*_*0*_ = 1.5 min^-1^. **(A)** In the left and right panels, we plot the steady state distributions of X (total protein) in the QSSA model and mechanistic model respectively starting from upper steady state. **(B)** In the left and right panels, we plot the steady state distributions of X (total protein) in the QSSA model and mechanistic model respectively starting from lower steady state. Using another random number sequence in the left and right panels, we plot the steady state distribution of X (total protein) in the QSSA model and mechanistic model respectively starting from **(C)** upper steady state **(D)** lower steady state.
(TIFF)

S4 Fig Sensitivity analysis of the parameters involved at *J*_*0*_ = 3 min^-1^. **(A)** K_C_ = 0.2 (*τ*_P_ = 700 min, *τ*_M_ = 35 min) and **(B)** K_C_ = 1.0 (*τ*_P_ = 700 min, *τ*_M_ = 7 min). For both cases sensitivity is measured on the basis of CVs of stochastic mean of X as a function of rate constants involved in QSSA model and mechanistic model. Here CV_X_ refers to the coefficient of variation in stochastic mean of X resulting due to parameter variation. It is divided by the CV of our standard stochastic model (Fig 3A) using our model-parameter set at K_C_ = 0.2. In all the cases parameters are increased individually at an amount of 10% of the model parameters keeping all other parameters constant.
(TIFF)

S5 Fig Noise (CV) level in X with increase in X population at various M_P_ population levels. **(A)** Deterministically similar QSSA and mechanistic models, deterministic mean of X (molecules) = 457.9 and M_P_ (molecules) = 46.3 at *J*_*0*_ = 7.5E-01 min^-1^. The solid lines represent stable steady states and dotted lines represent unstable steady states; *J*_*0*_ is the basal rate of M_P_ synthesis and acts as the bifurcation parameter. **(B)** Coefficient of variation of total protein (X) versus X abundance keeping deterministic mean of M_P_ = 46.3 molecules at *J*_*0*_ = 7.5E-01 min^-1^ with various K_C_. CVs of X are plotted at K_C_ = 1E-02, K_C_ = 1E-01, K_C_ = 2E-01 and K_C_ = 1.0 respectively. **(C)** Deterministically similar QSSA and mechanistic models, deterministic mean of X (molecules) = 457.9 and M_P_ (molecules) = 370.4 at *J*_*0*_ = 6 min^-1^. **(D)** Coefficient of variation of X versus X abundance keeping deterministic mean of M_P_ = 370.4 molecules at *J*_*0*_ = 6 min^-1^ with various K_C_. CVs of X are plotted at K_C_ = 1E-02, K_C_ = 1E-01, K_C_ = 2E-01 and K_C_ = 1.0 respectively. **(E)** Deterministically similar QSSA and mechanistic models with deterministic mean of X (molecules) = 457.9 and M_P_ (molecules) = 740.9 at *J*_*0*_ = 12 min^-1^. **(F)** Coefficient of variation of X versus X abundance keeping deterministic mean of M_P_ = 740.9 molecules at *J*_*0*_ = 12 min^-1^ with various K_C_. CVs of X are plotted at K_C_ = 1E-02, K_C_ = 1E-01, K_C_ = 2E-01 and K_C_ = 1.0 respectively. In all the cases *τ*_M_~7 min, *k*_*m*_ = 1E-01 min^-1^. Deviation between QSSA model and mechanistic model is more pronounced at higher K_C_.
(TIFF)

S6 Fig Mechanistic frameworks of different gene regulatory networks under consideration. **(A)** Gene regulatory network with positive and additional negative feedback motifs under consideration where G and G_a_ denote inactive and active states of gene, M_P_ denotes mRNA, P denotes protein, P_2_ is the dimer of protein. The protein (P) molecules form dimers P_2_. P_2_ binds to the promoter region of the inactive gene and activates G to G_a_ as well as P_2_ binds to the promoter region of another inactive gene G_s_ and activates G_s_ to G_as_. mRNAs (Y_M_) are synthesized at a basal rate *J*_*4*._ *k*_*m*_ and *k*_*p*_ are the degradation rates of mRNA of P (M_P_) and P and *k*_*ym*_ and *k*_*yp*_ are the degradation rates of mRNA of Y_P_ (Y_M_) and Y_P_. Y_P_ activates the degradation of protein P at *K*_*n*_ rate. **(B)** Gene regulatory network with double positive feedback motifs under consideration where G and G_a_ denote inactive and active states of gene, M_p_ denotes mRNA, P denotes protein, P_2_ is the dimer of protein. The protein P form dimers P_2_. P_2_ binds to the promoter region of the inactive gene and activates G to G_a_ as well as P_2_ binds to the promoter region of another inactive gene G_s_ and activates G_s_ to G_as_. mRNAs (Y_M_) are synthesized at a basal rate *J*_*4*._ *k*_*m*_ and *k*_*p*_ are the degradation rates of M_P_ and P and *k*_*ym*_ and *k*_*yp*_ are the degradation rates of Y_M_ and Y_P_. Y_P_ activates the synthesis of protein P at *K*_*n*_ rate.
(TIFF)

S7 Fig Deterministically similar QSSA and mechanistic models of gene regulatory networks with additional positive and negative feedback motifs. The solid lines represent stable steady states and dotted lines represent unstable steady states; *J*_*0*_ is the basal rate of M_P_ synthesis and acts as the bifurcation parameter. **(A)** In the left and right panels, we plot steady state population of X in QSSA and mechanistic models respectively. **(B)** In the left and right panels, we plot steady state population of Y_P_ in QSSA and mechanistic models respectively at K_C_ = 1E-02, K_S_ = 1E-02, *τ*_P_ = 700 min, *τ*_M_ = 7 min [S6 Table]. Values of *K*_*n*_, given in the plots, are in molecule^-1^min^-1^. Deterministic mean of X = 390.9 molecules, Y_P_ = 149.6 molecules, M_P_ = 174.2 molecules and Y_M_ = 162.3 molecules at *J*_*0*_ = 3 min^-1^ when *K*_*n*_ = 2.5E-07 molecule^-1^min^-1^. Deterministic mean of X = 457.9 molecules, Y_P_ = 161.1 molecules, M_P_ = 185.2 molecules and Y_M_ = 174.7 molecules at *J*_*0*_ = 3 min^-1^ when *K*_*n*_ = 0 molecule^-1^min^-1^.
(TIFF)

S8 FigPlot of CV of proteins and mRNAs versus ratio of half-lives (K_S_) of Y_M_ and Y_P_ as well as the absolute values of the half-lives of Y_M_ and Y_P_ at K_C_ = 1.0 in the module 1 with additional negative feedback on X.
**(A)** *τ*_P_ = 7 min, *τ*_M_ = 7 min and $\tau_{Y_{M}}$ = 7 min **(B)** *τ*_P_ = 700 min, *τ*_M_ = 700 min and $\tau_{Y_{M}}$ = 7 min. **(C)** Plots of CV of X, M_P_, Y_P_ and Y_M_ abundance in QSSA and mechanistic models versus absolute values of the half-lives of Y_M_ and Y_P_ at K_S_ = 1.0, keeping *τ*_P_ = 700 min, *τ*_M_ = 700 min. Values of $\tau_{Y_{M}}$ and $\tau_{Y_{P}}$ are given in minutes.(TIFF)

S9 Fig Deterministically similar QSSA and mechanistic models of gene regulatory network with double positive feedback motifs (additional positive feedback is working on X). The solid lines represent stable steady states and dotted lines represent unstable steady states; *J*_*0*_ is the basal rate of M_P_ synthesis and acts as the bifurcation parameter. **(A)** In the left and right panels, we plot steady state population of X in QSSA and mechanistic models respectively. **(B)** In the left and right panels, we plot steady state population of Y_P_ in QSSA and mechanistic models respectively at K_C_ = 1E-02, K_S_ = 1E-02, *τ*_P_ = 700 min, *τ*_M_ = 7 min [S8 Table]. Values of *K*_*n*_, given in the plots, are in min^-1^. Deterministic mean of X = 543.6 molecules, Y_P_ = 172.6 molecules, M_P_ = 196.3 molecules and Y_M_ = 187.1 molecules at *J*_*0*_ = 3 min^-1^ when *K*_*n*_ = 5E-05 min^-1^. Deterministic mean of X = 457.9 molecules, Y_P_ = 161.1 molecules, M_P_ = 185.2 molecules and Y_M_ = 174.7 molecules at *J*_*0*_ = 3 min^-1^ when *K*_*n*_ = 0 min^-1^.
(TIFF)

S10 FigPlot of CV of the total protein (X) and Y_P_ versus ratio of half-lives (K_S_) of Y_M_ and Y_P_ as well as the absolute values of the half-lives of Y_M_ and Y_P_ at K_C_ = 1E-02 in the module 2 with additional positive feedback on X.
**(A)** *τ*_P_ = 700 min, *τ*_M_ = 7 min and $\tau_{Y_{M}}$ = 7 min **(B)** *τ*_P_ = 10 min, *τ*_M_ = 1E-01 min and $\tau_{Y_{M}}$ = 7 min. **(C)** K_S_ = 1E-02, *τ*_P_ = 700 min and *τ*_M_ = 7 min. **(D)** K_S_ = 1.0, *τ*_P_ = 700 min and *τ*_M_ = 7 min. Values of $\tau_{Y_{M}}$ and $\tau_{Y_{P}}$ are given in minutes.(TIFF)

S11 FigPlot of CV of proteins and mRNAs versus ratio of half-lives (K_S_) of Y_M_ and Y_P_ as well as the absolute values of the half-lives of Y_M_ and Y_P_ at K_C_ = 1.0 in the module 2 with additional positive feedback on X.
**(A)** *τ*_P_ = 7 min, *τ*_M_ = 7 min and $\tau_{Y_{M}}$ = 7 min **(B)** *τ*_P_ = 700 min, *τ*_M_ = 700 min and $\tau_{Y_{M}}$ = 7 min. **(C)** Plots of CV of X, M_P_, Y_P_ and Y_M_ abundance in QSSA and mechanistic models versus absolute values of the half-lives of Y_M_ and Y_P_ at K_S_ = 1.0, keeping *τ*_P_ = 700 min, *τ*_M_ = 700 min. Values of $\tau_{Y_{M}}$ and $\tau_{Y_{P}}$ are given in minutes.(TIFF)

S1 Table Steady state analysis of the QSSA and mechanistic models. [S2 Fig].
(DOCX)

S2 Table (A) Stochastic results of Total protein (X) and mRNA (M_P_) at different K_C_ in QSSA model and mechanistic model. [Fig 3] (B) Parameters used in Fig 4. (DOCX)

S3 Table Parameters used in Fig 5. (DOCX)

S4 Table Parameters used in S5 Fig. (DOCX)

S5 Table Parameters used in Fig 6. (DOCX)

S6 Table Equations and parameters of the positive feedback with additional negative feedback on Protein level (Module 1). **[S7A and S7B Fig]**.
(DOCX)

S7 Table Parameters used in Fig 8 and S8 Fig. (DOCX)

S8 Table Equations and parameters of the additional positive feedback on Protein level (Module 2). [S9A and S9B Fig].
(DOCX)

S9 Table Parameters used in S10–S11 Figs. (DOCX)

S1 Text Mathematical modeling and stochastic version of different modules. (DOCX)

S2 Text Comparison of intrinsic noise as a function of the half-lives of proteins and mRNAs (for Module 2). (DOCX)
