# Supplementary figures and images for: Are Quasi-Steady-State Approximated Models Suitable for Quantifying Intrinsic Noise Accurately?
Source: PLoS One. 2015 Sep 1;10(9):e0136668. doi: 10.1371/journal.pone.0136668 (PMC4556639; doi:10.1371/journal.pone.0136668)

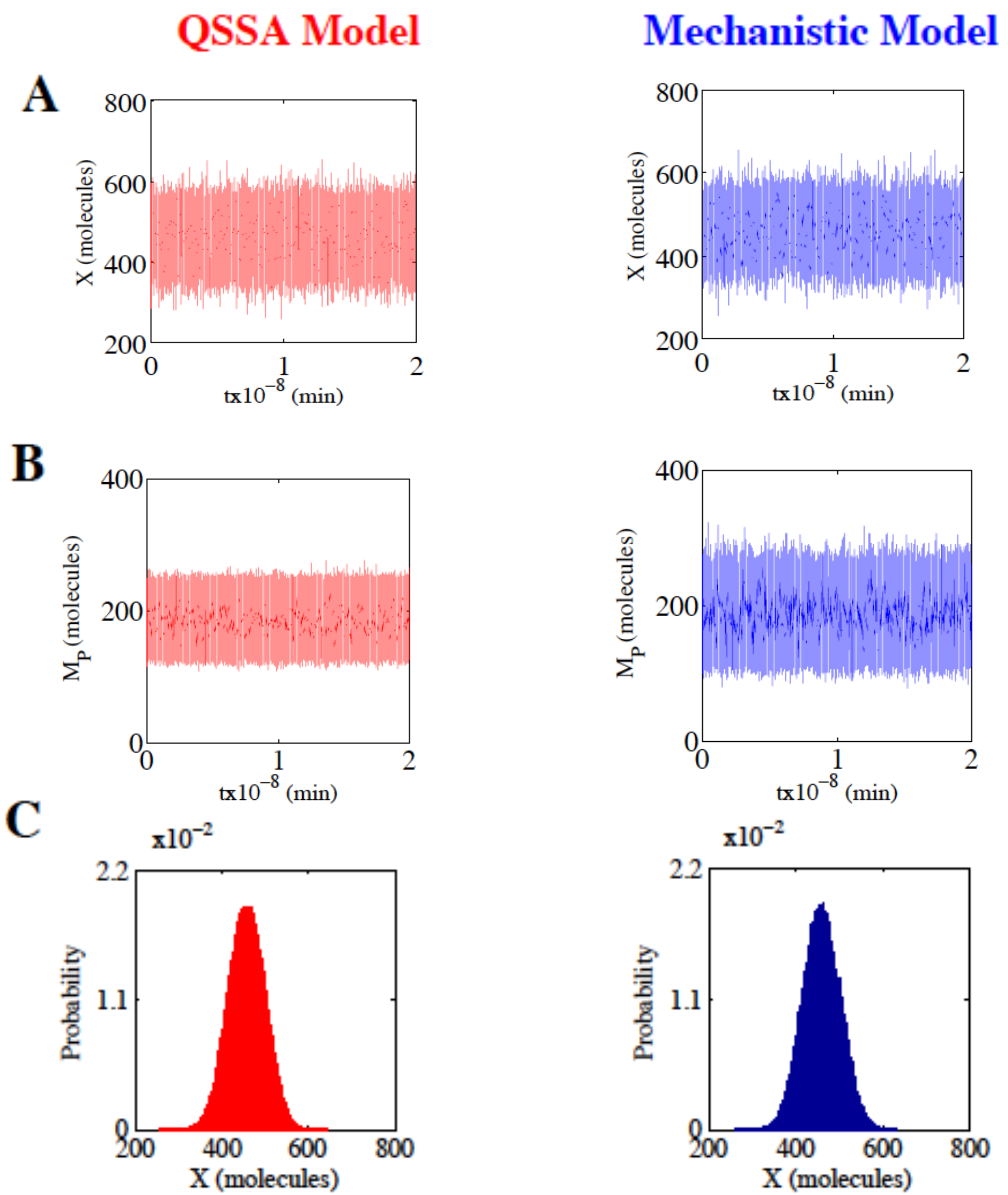

Supplement: S1 Fig — (A) In the left and right panels, we plot variation in population of X (total protein) with time in the QSSA model and mechanistic model respectively at J 0 = 3 min-1. (B) In the left and right panels, we plot variation of MP with time in the QSSA model and mechanistic model respectively at J 0 = 3 min-1. (C) Stochastic simulation based on Gillespie Algorithm using another random number sequence. In the left and right panels, we plot steady state distribution of X (total protein) in the QSSA model at J 0 = 3 min-1, mean = 457.3, standard deviation = 42.69, CV = 9.34% and steady state distribution of X (total protein) in the mechanistic model, mean = 461.4, standard deviation = 43.46, CV = 9.42% respectively. All the values (except CVs) are in number of molecules. (TIFF) [file pone.0136668.s001.tiff]

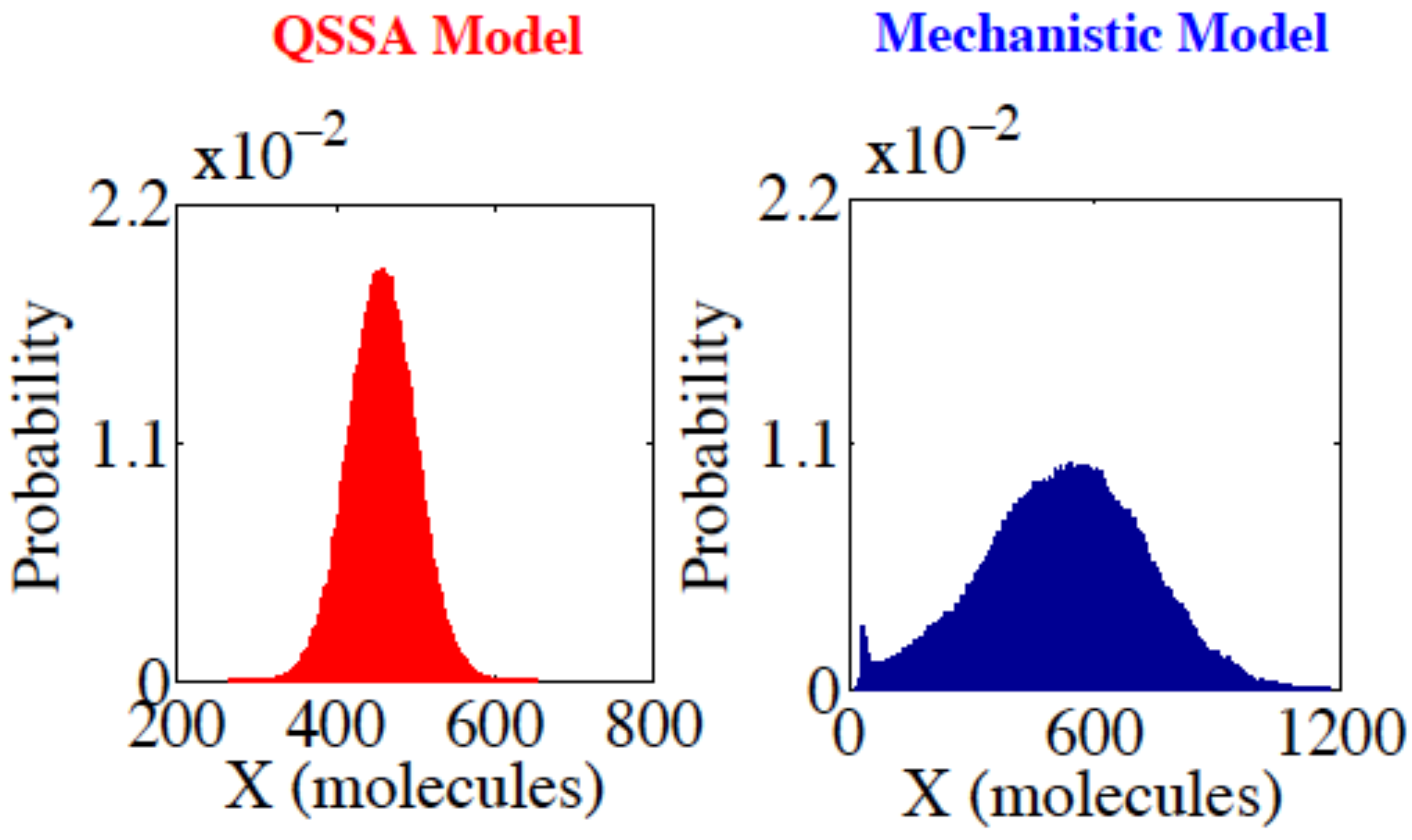

Supplement: S2 Fig — In the left panel, we plot steady state distribution of X (total protein) in the QSSA model at J 0 = 3 min-1, when k a and k d are 8E-03 min-1 and 5E-03 min-1, mean = 457.2, standard deviation = 42.98, CV = 9.4%. In the right panel, we plot steady state distribution of X (total protein) in the mechanistic model at J 0 = 3 min-1, when k a and k d are 8E-03 min-1 and 5E-03 min-1, mean = 523.7, standard deviation = 197.6, CV = 37.7%. All the values (except CVs) are in number of molecules. (TIFF) [file pone.0136668.s002.tiff]

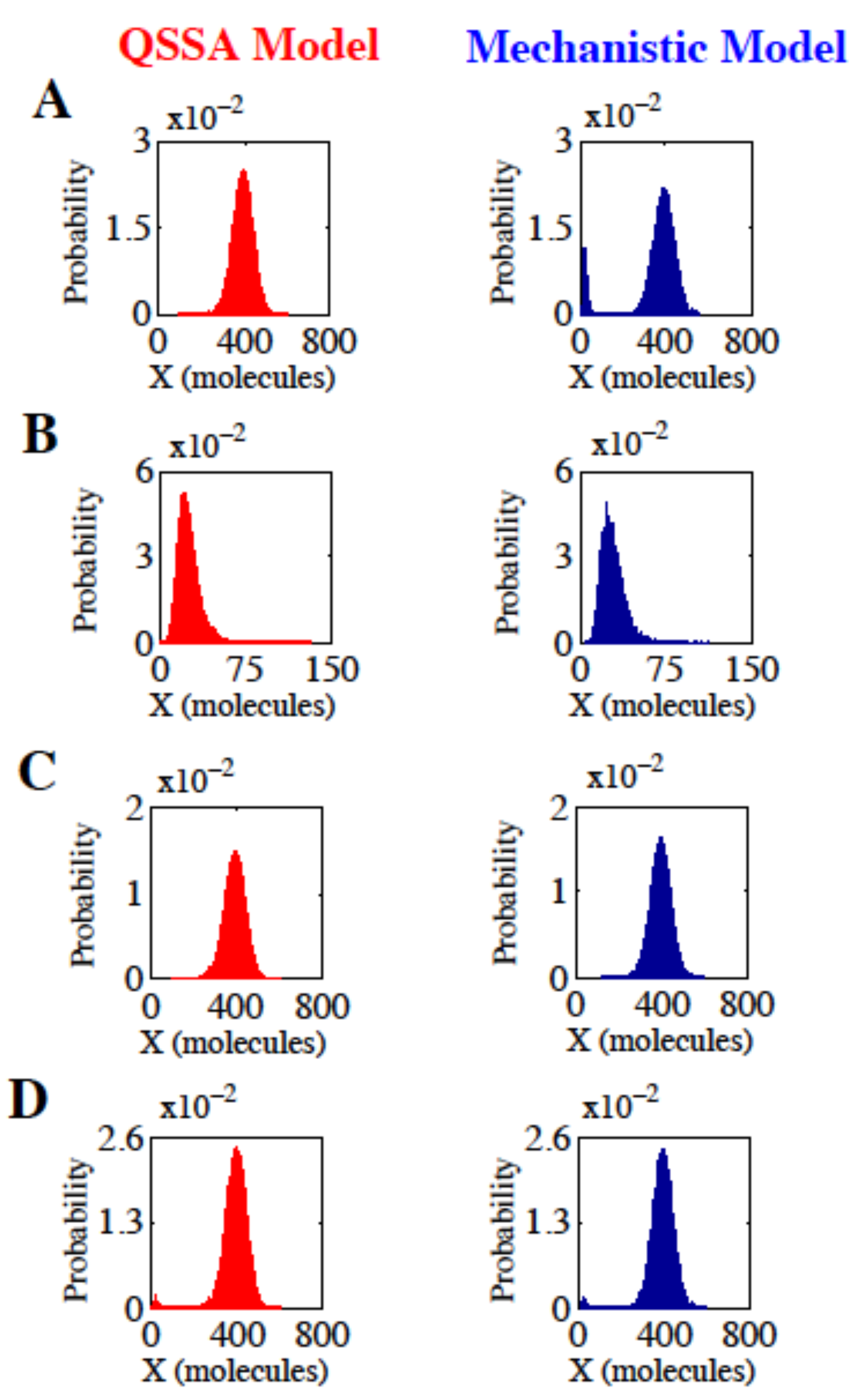

Supplement: S3 Fig — (A) In the left and right panels, we plot the steady state distributions of X (total protein) in the QSSA model and mechanistic model respectively starting from upper steady state. (B) In the left and right panels, we plot the steady state distributions of X (total protein) in the QSSA model and mechanistic model respectively starting from lower steady state. Using another random number sequence in the left and right panels, we plot the steady state distribution of X (total protein) in the QSSA model and mechanistic model respectively starting from (C) upper steady state (D) lower steady state. (TIFF) [file pone.0136668.s003.tiff]

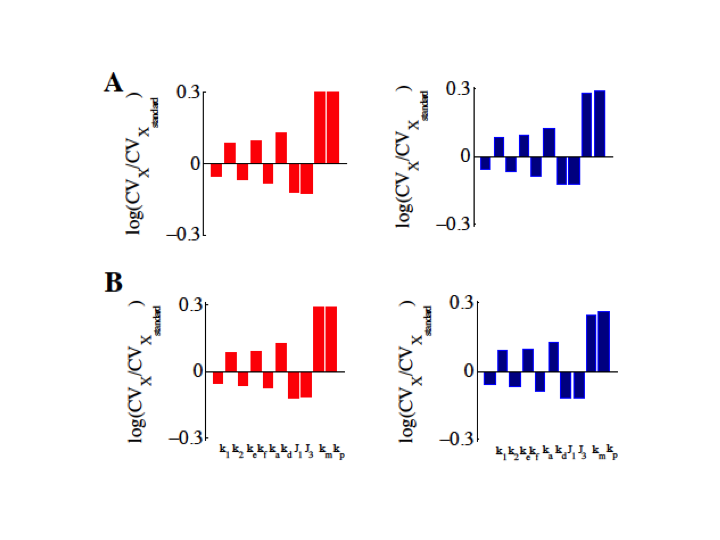

Supplement: S4 Fig — (A) KC = 0.2 (τ P = 700 min, τ M = 35 min) and (B) KC = 1.0 (τ P = 700 min, τ M = 7 min). For both cases sensitivity is measured on the basis of CVs of stochastic mean of X as a function of rate constants involved in QSSA model and mechanistic model. Here CVX refers to the coefficient of variation in stochastic mean of X resulting due to parameter variation. It is divided by the CV of our standard stochastic model (Fig 3A) using our model-parameter set at KC = 0.2. In all the cases parameters are increased individually at an amount of 10% of the model parameters keeping all other parameters constant. (TIFF) [file pone.0136668.s004.tiff]

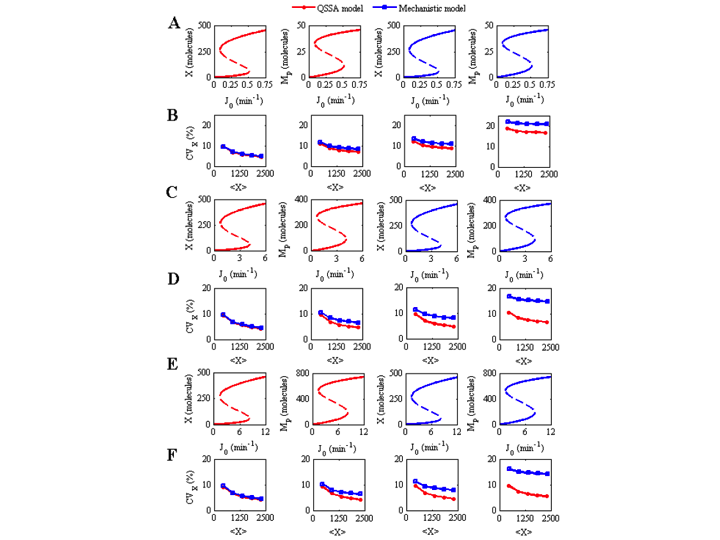

Supplement: S5 Fig — (A) Deterministically similar QSSA and mechanistic models, deterministic mean of X (molecules) = 457.9 and MP (molecules) = 46.3 at J 0 = 7.5E-01 min-1. The solid lines represent stable steady states and dotted lines represent unstable steady states; J 0 is the basal rate of MP synthesis and acts as the bifurcation parameter. (B) Coefficient of variation of total protein (X) versus X abundance keeping deterministic mean of MP = 46.3 molecules at J 0 = 7.5E-01 min-1 with various KC. CVs of X are plotted at KC = 1E-02, KC = 1E-01, KC = 2E-01 and KC = 1.0 respectively. (C) Deterministically similar QSSA and mechanistic models, deterministic mean of X (molecules) = 457.9 and MP (molecules) = 370.4 at J 0 = 6 min-1. (D) Coefficient of variation of X versus X abundance keeping deterministic mean of MP = 370.4 molecules at J 0 = 6 min-1 with various KC. CVs of X are plotted at KC = 1E-02, KC = 1E-01, KC = 2E-01 and KC = 1.0 respectively. (E) Deterministically similar QSSA and mechanistic models with deterministic mean of X (molecules) = 457.9 and MP (molecules) = 740.9 at J 0 = 12 min-1. (F) Coefficient of variation of X versus X abundance keeping deterministic mean of MP = 740.9 molecules at J 0 = 12 min-1 with various KC. CVs of X are plotted at KC = 1E-02, KC = 1E-01, KC = 2E-01 and KC = 1.0 respectively. In all the cases τ M~7 min, k m = 1E-01 min-1. Deviation between QSSA model and mechanistic model is more pronounced at higher KC. (TIFF) [file pone.0136668.s005.tiff]

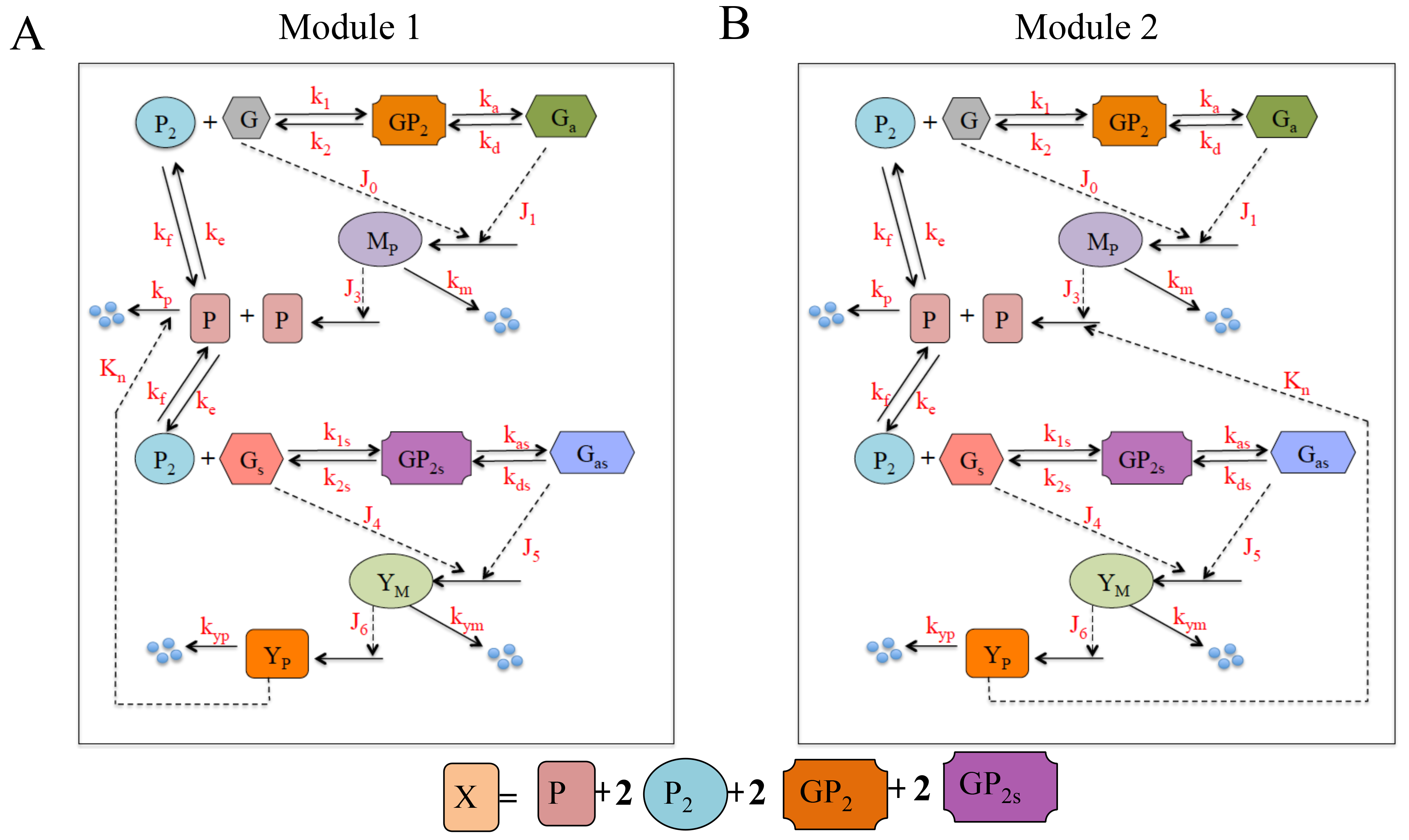

Supplement: S6 Fig — (A) Gene regulatory network with positive and additional negative feedback motifs under consideration where G and Ga denote inactive and active states of gene, MP denotes mRNA, P denotes protein, P2 is the dimer of protein. The protein (P) molecules form dimers P2. P2 binds to the promoter region of the inactive gene and activates G to Ga as well as P2 binds to the promoter region of another inactive gene Gs and activates Gs to Gas. mRNAs (YM) are synthesized at a basal rate J 4. k m and k p are the degradation rates of mRNA of P (MP) and P and k ym and k yp are the degradation rates of mRNA of YP (YM) and YP. YP activates the degradation of protein P at K n rate. (B) Gene regulatory network with double positive feedback motifs under consideration where G and Ga denote inactive and active states of gene, Mp denotes mRNA, P denotes protein, P2 is the dimer of protein. The protein P form dimers P2. P2 binds to the promoter region of the inactive gene and activates G to Ga as well as P2 binds to the promoter region of another inactive gene Gs and activates Gs to Gas. mRNAs (YM) are synthesized at a basal rate J 4. k m and k p are the degradation rates of MP and P and k ym and k yp are the degradation rates of YM and YP. YP activates the synthesis of protein P at K n rate. (TIFF) [file pone.0136668.s006.tiff]

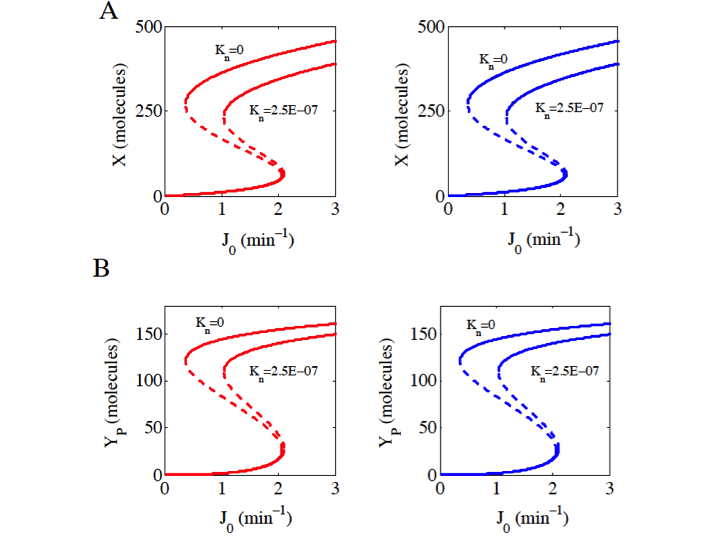

Supplement: S7 Fig — The solid lines represent stable steady states and dotted lines represent unstable steady states; J 0 is the basal rate of MP synthesis and acts as the bifurcation parameter. (A) In the left and right panels, we plot steady state population of X in QSSA and mechanistic models respectively. (B) In the left and right panels, we plot steady state population of YP in QSSA and mechanistic models respectively at KC = 1E-02, KS = 1E-02, τ P = 700 min, τ M = 7 min [S6 Table]. Values of K n, given in the plots, are in molecule-1min-1. Deterministic mean of X = 390.9 molecules, YP = 149.6 molecules, MP = 174.2 molecules and YM = 162.3 molecules at J 0 = 3 min-1 when K n = 2.5E-07 molecule-1min-1. Deterministic mean of X = 457.9 molecules, YP = 161.1 molecules, MP = 185.2 molecules and YM = 174.7 molecules at J 0 = 3 min-1 when K n = 0 molecule-1min-1. (TIFF) [file pone.0136668.s007.tiff]

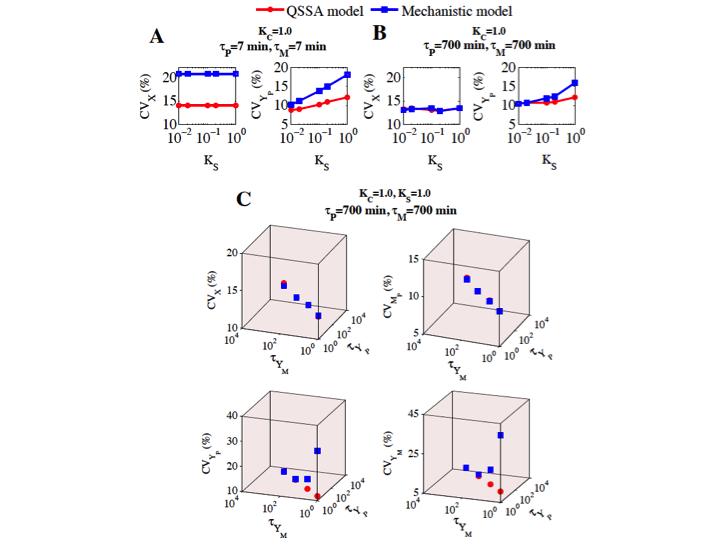

Supplement: S8 Fig — (A) τ P = 7 min, τ M = 7 min and τYM = 7 min (B) τ P = 700 min, τ M = 700 min and τYM = 7 min. (C) Plots of CV of X, MP, YP and YM abundance in QSSA and mechanistic models versus absolute values of the half-lives of YM and YP at KS = 1.0, keeping τ P = 700 min, τ M = 700 min. Values of τYM and τYP are given in minutes. (TIFF) [file pone.0136668.s008.tiff]

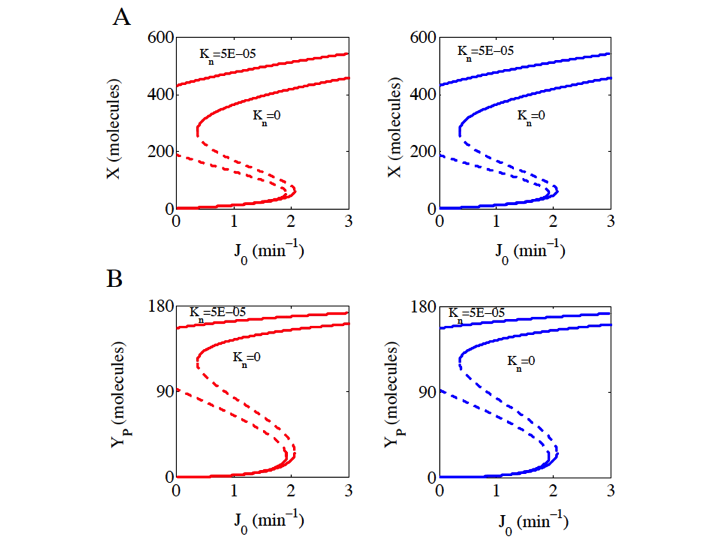

Supplement: S9 Fig — The solid lines represent stable steady states and dotted lines represent unstable steady states; J 0 is the basal rate of MP synthesis and acts as the bifurcation parameter. (A) In the left and right panels, we plot steady state population of X in QSSA and mechanistic models respectively. (B) In the left and right panels, we plot steady state population of YP in QSSA and mechanistic models respectively at KC = 1E-02, KS = 1E-02, τ P = 700 min, τ M = 7 min [S8 Table]. Values of K n, given in the plots, are in min-1. Deterministic mean of X = 543.6 molecules, YP = 172.6 molecules, MP = 196.3 molecules and YM = 187.1 molecules at J 0 = 3 min-1 when K n = 5E-05 min-1. Deterministic mean of X = 457.9 molecules, YP = 161.1 molecules, MP = 185.2 molecules and YM = 174.7 molecules at J 0 = 3 min-1 when K n = 0 min-1. (TIFF) [file pone.0136668.s009.tiff]

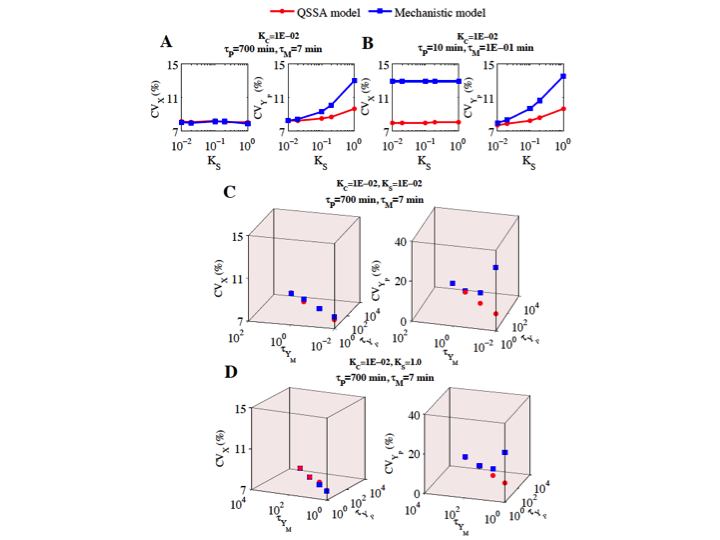

Supplement: S10 Fig — (A) τ P = 700 min, τ M = 7 min and τYM = 7 min (B) τ P = 10 min, τ M = 1E-01 min and τYM = 7 min. (C) KS = 1E-02, τ P = 700 min and τ M = 7 min. (D) KS = 1.0, τ P = 700 min and τ M = 7 min. Values of τYM and τYP are given in minutes. (TIFF) [file pone.0136668.s010.tiff]

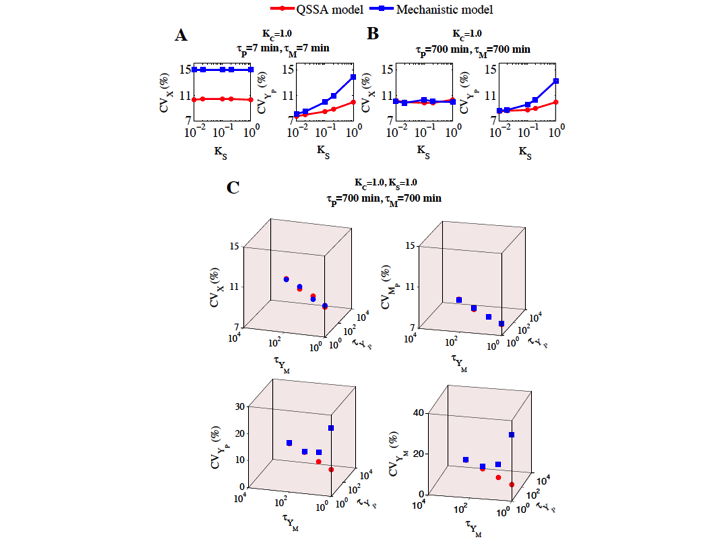

Supplement: S11 Fig — (A) τ P = 7 min, τ M = 7 min and τYM = 7 min (B) τ P = 700 min, τ M = 700 min and τYM = 7 min. (C) Plots of CV of X, MP, YP and YM abundance in QSSA and mechanistic models versus absolute values of the half-lives of YM and YP at KS = 1.0, keeping τ P = 700 min, τ M = 700 min. Values of τYM and τYP are given in minutes. (TIFF) [file pone.0136668.s011.tiff]
