# Supplementary material for: Are Quasi-Steady-State Approximated Models Suitable for Quantifying Intrinsic Noise Accurately?
Source: PLoS One. 2015 Sep 1;10(9):e0136668. doi: 10.1371/journal.pone.0136668 (PMC4556639; doi:10.1371/journal.pone.0136668)
Supplement: S1 Table — [S2 Fig]. (DOCX) [file pone.0136668.s012.docx]

**S1 Table. Steady state analysis of the QSSA and mechanistic models. [S2 Fig.]**

QSSA model Mechanistic model

| **Model No.** | ***k_a_***  **_(min_^-1^_)_** | ***k_d_***  **_(min_^-1^_)_** | **<X>**  **_(molecules)_** | **X_Std_**  **_(molecules)_** | **X_CV_ (%)** | **<X>**  **_(molecules)_** | **X_Std_**  **_(molecules)_** | **X_CV_**  **(%)** |
| --- | --- | --- | --- | --- | --- | --- | --- | --- |
| 1 | 8.0E-03 | 5.0E-03 | 457.2 | 42.98 | 9.4 | 523.7 | 197.6 | 37.7 |
| 2 | 8.0E-02 | 5.0E-02 | 457.2 | 42.98 | 9.4 | 469.2 | 81.4 | 17.4 |
| 3 | 8.0 | 5.0 | 457.2 | 42.98 | 9.4 | 462.3 | 43.54 | 9.42 |
