# Supplementary material for: Are Quasi-Steady-State Approximated Models Suitable for Quantifying Intrinsic Noise Accurately?
Source: PLoS One. 2015 Sep 1;10(9):e0136668. doi: 10.1371/journal.pone.0136668 (PMC4556639; doi:10.1371/journal.pone.0136668)
Supplement: S2 Table — (DOCX) [file pone.0136668.s013.docx]

**S2 Table.**

**(A) Stochastic results of Total protein (X) and mRNA (M_P_) at different K_C_ in QSSA model and mechanistic model. [Figure 3]**

QSSA model Mechanistic model

| **Model No.** | **ζ_P_**  **_(min)_** | **K_C_** | **<X>**  **_(molecules)_** | **X_Std_**  **_(molecules)_** | **X_CV_ (%)** | **<X>**  **_(molecules)_** | **X_Std_**  **_(molecules)_** | **X_CV_**  **(%)** |
| --- | --- | --- | --- | --- | --- | --- | --- | --- |
| 1 | 1 | 7 | 458.8 | 75.96 | 16.56 | 488.9 | 135.15 | 27.65 |
| 2 | 7 | 1 | 457.9 | 54.91 | 11.99 | 470.1 | 82.68 | 17.59 |
| 3 | 21 | 0.33 | 457.4 | 47.73 | 10.43 | 465.2 | 61.10 | 13.13 |
| 4 | 35 | 0.2 | 457.6 | 45.87 | 10.02 | 464.2 | 54.74 | 11.79 |
| 5 | 70 | 0.1 | 457.4 | 44.47 | 9.72 | 462.6 | 49.33 | 10.66 |
| 6 | 150 | 0.046 | 457.3 | 43.61 | 9.54 | 462.8 | 45.67 | 9.87 |
| 7 | 210 | 0.033 | 457.3 | 43.35 | 9.48 | 462.7 | 44.87 | 9.7 |
| 8 | 280 | 0.025 | 457.9 | 43.16 | 9.42 | 462.5 | 44.52 | 9.63 |
| 9 | 350 | 0.02 | 457.2 | 43.05 | 9.42 | 462.1 | 43.99 | 9.52 |
| 10 | 700 | 0.01 | 457.2 | 42.98 | 9.4 | 462.3 | 43.54 | 9.42 |

QSSA model Mechanistic model

| **Model No.** | **ζ_P_**  **_(min)_** | **K_C_** | **<M_P_>**  **_(molecules)_** | **M_P Std_**  **_(molecules)_** | **M_P CV_ (%)** | **<M_P_>**  **_(molecules)_** | **M_P Std_**  **_(molecules)_** | **M_P CV_ (%)** |
| --- | --- | --- | --- | --- | --- | --- | --- | --- |
| 1 | 1 | 7 | 184.7 | 19.38 | 10.49 | 190.1 | 36.82 | 19.37 |
| 2 | 7 | 1 | 185.1 | 16.45 | 8.89 | 186.9 | 30.50 | 16.32 |
| 3 | 21 | 0.33 | 185.1 | 15.58 | 8.41 | 186.1 | 28.52 | 15.32 |
| 4 | 35 | 0.2 | 185.2 | 15.38 | 8.3 | 185.9 | 28.01 | 15.06 |
| 5 | 70 | 0.1 | 185.2 | 15.22 | 8.22 | 185.8 | 27.71 | 14.91 |
| 6 | 150 | 0.046 | 185.2 | 15.13 | 8.17 | 185.8 | 27.39 | 14.74 |
| 7 | 210 | 0.033 | 185.2 | 15.09 | 8.15 | 185.7 | 27.35 | 14.72 |
| 8 | 280 | 0.025 | 185.3 | 15.07 | 8.13 | 185.7 | 27.42 | 14.76 |
| 9 | 350 | 0.02 | 185.2 | 15.07 | 8.14 | 185.7 | 27.38 | 14.75 |
| 10 | 700 | 0.01 | 185.2 | 15.05 | 8.13 | 185.6 | 27.26 | 14.68 |

**(B) Parameters used in Fig 4.**

**Figure 4 (A) and Figure 4(D):**

**K_C_=1E-02**

| **Model No.** | ***k_p_***  (min^-1^) | ***k_m_***  (min^-1^) | ***J_3_***  (min^-1^) | ***J_1_***  (min^-1^) | ***J_0_***  (min^-1^) |
| --- | --- | --- | --- | --- | --- |
| 1 | 1.0E-04 | 1.0E-02 | 9.22E-05 | 4.3838 | 3.0E-01 |
| 2 | 1.0E-03 | 1.0E-01 | 9.22E-04 | 43.838 | 3.0 |
| 3 | 7.0E-03 | 7.0E-01 | 6.46E-03 | 306.9 | 21.0 |
| 4 | 7.0E-02 | 7.0 | 6.46E-02 | 3069.0 | 210.0 |
| 5 | 7.0E-01 | 70.0 | 6.46E-01 | 30690.0 | 2100.0 |

**Figure 4 (B) and Figure 4(E):**

**K_C_=1.0**

| **Model No.** | ***k_p_***  (min^-1^) | ***k_m_***  (min^-1^) | ***J_3_***  (min^-1^) | ***J_1_***  (min^-1^) | ***J_0_***  (min^-1^) |
| --- | --- | --- | --- | --- | --- |
| 1 | 5.0E-04 | 5.0E-04 | 4.61E-04 | 2.1919E-01 | 1.5E-02 |
| 2 | 1.0E-03 | 1.0E-03 | 9.22E-04 | 43.838E-02 | 3.0E-02 |
| 3 | 1.0E-02 | 1.0E-02 | 9.22E-03 | 4.3838 | 3.0E-01 |
| 4 | 1.0E-01 | 1.0E-01 | 9.22E-02 | 43.838 | 3.0 |
| 5 | 7.0E-01 | 7.0E-01 | 6.46E-01 | 306.9 | 21.0 |

**Figure 4 (C) and Figure 4(F):**

**K_C_=100.0**

| **Model No.** | ***k_p_***  (min^-1^) | ***k_m_***  (min^-1^) | ***J_3_***  (min^-1^) | ***J_1_***  (min^-1^) | ***J_0_***  (min^-1^) |
| --- | --- | --- | --- | --- | --- |
| 1 | 5.0E-02 | 5.0E-04 | 4.61E-02 | 2.1919E-01 | 1.5E-02 |
| 2 | 1.0E-01 | 1.0E-03 | 9.22E-02 | 43.838E-02 | 3.0E-02 |
| 3 | 7.0E-01 | 7.0E-03 | 6.46E-01 | 3.069 | 2.1E-01 |
| 4 | 7.0 | 7.0E-02 | 6.46 | 30.69 | 2.1 |
| 5 | 70.0 | 7.0E-01 | 64.55 | 306.9 | 21.0 |
