# Supplementary material for: Are Quasi-Steady-State Approximated Models Suitable for Quantifying Intrinsic Noise Accurately?
Source: PLoS One. 2015 Sep 1;10(9):e0136668. doi: 10.1371/journal.pone.0136668 (PMC4556639; doi:10.1371/journal.pone.0136668)
Supplement: S3 Table — (DOCX) [file pone.0136668.s014.docx]

**S3 Table. Parameters used in Fig 5.**

**Figure 5 (A):**

**K_C_=1E-02, *k_m_*=1E-01 min^-1^, *J_1_*=43.838 min^-1^, *J_0_*=3 min^-1^**

| **Model No.** | ***k_p_***  (min^-1^) | ***J_3_***  (min^-1^) | ***k_1_***  (molecule^-1^min^-1^) | ***k_e_***  (molecule^-1^min^-1^) |
| --- | --- | --- | --- | --- |
| 1 | 1.0E-03 | 9.22E-04 | 4.0E-01 | 4.0E-01 |
| 2 | 1.0E-03 | 1.85E-03 | 2.0E-01 | 2.0E-01 |
| 3 | 1.0E-03 | 2.77E-03 | 1.33E-01 | 1.33E-01 |
| 4 | 1.0E-03 | 3.69E-03 | 1.0E-01 | 1.0E-01 |
| 5 | 1.0E-03 | 4.61E-03 | 8.0E-02 | 8.0E-02 |

**Figure 5 (B):**

**K_C_ =1E-01, *k_m_*=1E-01 min^-1^, *J_1_*=43.838 min^-1^, *J_0_*=3 min^-1^**

| **Model No.** | ***k_p_***  (min^-1^) | ***J_3_***  (min^-1^) | ***k_1_***  (molecule^-1^min^-1^) | ***k_e_***  (molecule^-1^min^-1^) |
| --- | --- | --- | --- | --- |
| 1 | 1.0E-02 | 9.22E-03 | 4.0E-01 | 4.0E-01 |
| 2 | 1.0E-02 | 1.85E-02 | 2.0E-01 | 2.0E-01 |
| 3 | 1.0E-02 | 2.77E-02 | 1.33E-01 | 1.33E-01 |
| 4 | 1.0E-02 | 3.69E-02 | 1.0E-01 | 1.0E-01 |
| 5 | 1.0E-02 | 4.61E-02 | 8.0E-02 | 8.0E-02 |

**Figure 5 (C):**

**K_C_ =1.0, *k_m_*=1E-01 min^-1^, *J_1_*=43.838 min^-1^, *J_0_*=3 min^-1^**

| **Model No.** | ***k_p_***  (min^-1^) | ***J_3_***  (min^-1^) | ***k_1_***  (molecule^-1^min^-1^) | ***k_e_***  (molecule^-1^min^-1^) |
| --- | --- | --- | --- | --- |
| 1 | 1.0E-01 | 9.22E-02 | 4.0E-01 | 4.0E-01 |
| 2 | 1.0E-01 | 1.85E-01 | 2.0E-01 | 2.0E-01 |
| 3 | 1.0E-01 | 2.77E-01 | 1.33E-01 | 1.33E-01 |
| 4 | 1.0E-01 | 3.69E-01 | 1.0E-01 | 1.0E-01 |
| 5 | 1.0E-01 | 4.61E-01 | 8.0E-02 | 8.0E-02 |

**Figure 5 (D):**

**K_C_ =100.0, *k_m_*=1E-01 min^-1^, *J_1_*=43.838 min^-1^, *J_0_*=3 min^-1^**

| **Model No.** | ***k_p_***  (min^-1^) | ***J_3_***  (min^-1^) | ***k_1_***  (molecule^-1^min^-1^) | ***k_e_***  (molecule^-1^min^-1^) |
| --- | --- | --- | --- | --- |
| 1 | 10.0 | 9.22 | 4.0E-01 | 4.0E-01 |
| 2 | 10.0 | 18.45 | 2.0E-01 | 2.0E-01 |
| 3 | 10.0 | 27.67 | 1.33E-01 | 1.33E-01 |
| 4 | 10.0 | 36.89 | 1.0E-01 | 1.0E-01 |
| 5 | 10.0 | 46.11 | 8.0E-02 | 8.0E-02 |

**Figure 5 (E):**

**K_C_=1E-02, *k_m_*=7.0 min^-1^, *J_1_*=3069.0 min^-1^, *J_0_*=210 min^-1^**

| **Model No.** | ***k_p_***  (min^-1^) | ***J_3_***  (min^-1^) | ***k_1_***  (molecule^-1^min^-1^) | ***k_e_***  (molecule^-1^min^-1^) |
| --- | --- | --- | --- | --- |
| 1 | 7.0E-02 | 6.46E-02 | 4.0E-01 | 4.0E-01 |
| 2 | 7.0E-02 | 1.29E-01 | 2.0E-01 | 2.0E-01 |
| 3 | 7.0E-02 | 1.94E-01 | 1.33E-01 | 1.33E-01 |
| 4 | 7.0E-02 | 2.58E-01 | 1.0E-01 | 1.0E-01 |
| 5 | 7.0E-02 | 3.23E-01 | 8.0E-02 | 8.0E-02 |

**Figure 5 (F):**

**K_C_=100.0, *k_m_*=1E-03 min^-1^, *J_1_*=43.838E-02 min^-1^, *J_0_*=3.0E-02 min^-1^**

| **Model No.** | ***k_p_***  (min^-1^) | ***J_3_***  (min^-1^) | ***k_1_***  (molecule^-1^min^-1^) | ***k_e_***  (molecule^-1^min^-1^) |
| --- | --- | --- | --- | --- |
| 1 | 1.0E-01 | 9.22E-02 | 4.0E-01 | 4.0E-01 |
| 2 | 1.0E-01 | 18.45E-02 | 2.0E-01 | 2.0E-01 |
| 3 | 1.0E-01 | 27.67E-02 | 1.33E-01 | 1.33E-01 |
| 4 | 1.0E-01 | 36.89E-02 | 1.0E-01 | 1.0E-01 |
| 5 | 1.0E-01 | 46.11E-02 | 8.0E-02 | 8.0E-02 |

All other parameters were same as Table 2**_._**
