# Supplementary material for: Are Quasi-Steady-State Approximated Models Suitable for Quantifying Intrinsic Noise Accurately?
Source: PLoS One. 2015 Sep 1;10(9):e0136668. doi: 10.1371/journal.pone.0136668 (PMC4556639; doi:10.1371/journal.pone.0136668)
Supplement: S4 Table — (DOCX) [file pone.0136668.s015.docx]

**S4 Table. Parameters used in S5 Fig.**

**Figure S5 (B):**

**K_C_ =1E-02, *J_0_*=7.5E-01 min^-1^**

| **Model No.** | ***k_p_***  (min^-1^) | ***J_1_***  (min^-1^) | ***J_3_***  (min^-1^) | ***k_1_=k_e_***  (molecule^-1^min^-1^) |
| --- | --- | --- | --- | --- |
| 1 | 1.0E-03 | 10.96 | 3.69E-03 | 4.0E-01 |
| 2 | 1.0E-03 | 10.96 | 7.38E-03 | 2.0E-01 |
| 3 | 1.0E-03 | 10.96 | 1.11E-02 | 1.33E-01 |
| 4 | 1.0E-03 | 10.96 | 1.48E-02 | 1.0E-01 |
| 5 | 1.0E-03 | 10.96 | 1.85E-02 | 8.0E-02 |

**Figure S5 (B):**

**K_C_ =1E-01, *J_0_*=7.5E-01 min^-1^**

| **Model No.** | ***k_p_***  (min^-1^) | ***J_1_***  (min^-1^) | ***J_3_***  (min^-1^) | ***k_1_=k_e_***  (molecule^-1^min^-1^) |
| --- | --- | --- | --- | --- |
| 1 | 1.0E-02 | 10.96 | 3.69E-02 | 4.0E-01 |
| 2 | 1.0E-02 | 10.96 | 7.38E-02 | 2.0E-01 |
| 3 | 1.0E-02 | 10.96 | 1.11E-01 | 1.33E-01 |
| 4 | 1.0E-02 | 10.96 | 1.48E-01 | 1.0E-01 |
| 5 | 1.0E-02 | 10.96 | 1.85E-01 | 8.0E-02 |

**Figure S5 (B):**

**K_C_ =2E-01 *J_0_*=7.5E-01 min^-1^**

| **Model No.** | ***k_p_***  (min^-1^) | ***J_1_***  (min^-1^) | ***J_3_***  (min^-1^) | ***k_1_=k_e_***  (molecule^-1^min^-1^) |
| --- | --- | --- | --- | --- |
| 1 | 2.0E-02 | 10.96 | 7.38E-02 | 4.0E-01 |
| 2 | 2.0E-02 | 10.96 | 1.48E-01 | 2.0E-01 |
| 3 | 2.0E-02 | 10.96 | 2.21E-01 | 1.33E-01 |
| 4 | 2.0E-02 | 10.96 | 2.95E-01 | 1.0E-01 |
| 5 | 2.0E-02 | 10.96 | 3.69E-01 | 8.0E-02 |

**Figure S5 (B):**

**K_C_ =1.0, *J_0_*=7.5E-01 min^-1^**

| **Model No.** | ***k_p_***  (min^-1^) | ***J_1_***  (min^-1^) | ***J_3_***  (min^-1^) | ***k_1_=k_e_***  (molecule^-1^min^-1^) |
| --- | --- | --- | --- | --- |
| 1 | 1.0E-01 | 10.96 | 3.69E-01 | 4.0E-01 |
| 2 | 1.0E-01 | 10.96 | 7.38E-01 | 2.0E-01 |
| 3 | 1.0E-01 | 10.96 | 1.11 | 1.33E-01 |
| 4 | 1.0E-01 | 10.96 | 1.48 | 1.0E-01 |
| 5 | 1.0E-01 | 10.96 | 1.85 | 8.0E-02 |

All other parameters were same as Table 2**_._**

**Figure S5 (D):**

**K_C_ =1E-02, *J_0_*=6.0 min^-1^**

| **Model No.** | ***k_p_***  (min^-1^) | ***J_1_***  (min^-1^) | ***J_3_***  (min^-1^) | ***k_1_=k_e_***  (molecule^-1^min^-1^) |
| --- | --- | --- | --- | --- |
| 1 | 1.0E-03 | 87.676 | 4.61E-04 | 4.0E-01 |
| 2 | 1.0E-03 | 87.676 | 9.22E-04 | 2.0E-01 |
| 3 | 1.0E-03 | 87.676 | 1.38E-03 | 1.33E-01 |
| 4 | 1.0E-03 | 87.676 | 1.85E-03 | 1.0E-01 |
| 5 | 1.0E-03 | 87.676 | 2.31E-03 | 8.0E-02 |

**Figure S5 (D):**

**K_C_ =1E-01, *J_0_*=6.0 min^-1^**

| **Model No.** | ***k_p_***  (min^-1^) | ***J_1_***  (min^-1^) | ***J_3_***  (min^-1^) | ***k_1_=k_e_***  (molecule^-1^min^-1^) |
| --- | --- | --- | --- | --- |
| 1 | 1.0E-02 | 87.676 | 4.61E-03 | 4.0E-01 |
| 2 | 1.0E-02 | 87.676 | 9.22E-03 | 2.0E-01 |
| 3 | 1.0E-02 | 87.676 | 1.38E-02 | 1.33E-01 |
| 4 | 1.0E-02 | 87.676 | 1.85E-02 | 1.0E-01 |
| 5 | 1.0E-02 | 87.676 | 2.31E-02 | 8.0E-02 |

**Figure S5 (D):**

**K_C_ =2E-01, *J_0_*=6.0 min^-1^**

| **Model No.** | ***k_p_***  (min^-1^) | ***J_1_***  (min^-1^) | ***J_3_***  (min^-1^) | ***k_1_=k_e_***  (molecule^-1^min^-1^) |
| --- | --- | --- | --- | --- |
| 1 | 2.0E-02 | 87.676 | 9.22E-03 | 4.0E-01 |
| 2 | 2.0E-02 | 87.676 | 1.85E-02 | 2.0E-01 |
| 3 | 2.0E-02 | 87.676 | 2.77E-02 | 1.33E-01 |
| 4 | 2.0E-02 | 87.676 | 3.69E-02 | 1.0E-01 |
| 5 | 2.0E-02 | 87.676 | 4.61E-02 | 8.0E-02 |

**Figure S5 (D):**

**K_C_ =1.0, *J_0_*=6.0 min^-1^**

| **Model No.** | ***k_p_***  (min^-1^) | ***J_1_***  (min^-1^) | ***J_3_***  (min^-1^) | ***k_1_=k_e_***  (molecule^-1^min^-1^) |
| --- | --- | --- | --- | --- |
| 1 | 1.0E-01 | 87.676 | 4.61E-02 | 4.0E-01 |
| 2 | 1.0E-01 | 87.676 | 9.22E-02 | 2.0E-01 |
| 3 | 1.0E-01 | 87.676 | 1.38E-01 | 1.33E-01 |
| 4 | 1.0E-01 | 87.676 | 1.85E-01 | 1.0E-01 |
| 5 | 1.0E-01 | 87.676 | 2.31E-01 | 8.0E-02 |

All other parameters were same as Table 2**_._**

**Figure S5 (F):**

**K_C_ =1E-02, *J_0_*=12.0 min^-1^**

| **Model No.** | ***k_p_***  (min^-1^) | ***J_1_***  (min^-1^) | ***J_3_***  (min^-1^) | ***k_1_=k_e_***  (molecule^-1^min^-1^) |
| --- | --- | --- | --- | --- |
| 1 | 1.0E-03 | 175.352 | 2.31E-04 | 4.0E-01 |
| 2 | 1.0E-03 | 175.352 | 4.61E-04 | 2.0E-01 |
| 3 | 1.0E-03 | 175.352 | 6.92E-04 | 1.33E-01 |
| 4 | 1.0E-03 | 175.352 | 9.22E-04 | 1.0E-01 |
| 5 | 1.0E-03 | 175.352 | 1.15E-03 | 8.0E-02 |

**Figure S5 (F):**

**K_C_ =1E-01, *J_0_*=12.0 min^-1^**

| **Model No.** | ***k_p_***  (min^-1^) | ***J_1_***  (min^-1^) | ***J_3_***  (min^-1^) | ***k_1_=k_e_***  (molecule^-1^min^-1^) |
| --- | --- | --- | --- | --- |
| 1 | 1.0E-02 | 175.352 | 2.31E-03 | 4.0E-01 |
| 2 | 1.0E-02 | 175.352 | 4.61E-03 | 2.0E-01 |
| 3 | 1.0E-02 | 175.352 | 6.92E-03 | 1.33E-01 |
| 4 | 1.0E-02 | 175.352 | 9.22E-03 | 1.0E-01 |
| 5 | 1.0E-02 | 175.352 | 1.15E-02 | 8.0E-02 |

**Figure S5 (F):**

**K_C_ =2E-01, *J_0_*=12.0 min^-1^**

| **Model No.** | ***k_p_***  (min^-1^) | ***J_1_***  (min^-1^) | ***J_3_***  (min^-1^) | ***k_1_=k_e_***  (molecule^-1^min^-1^) |
| --- | --- | --- | --- | --- |
| 1 | 2.0E-02 | 175.352 | 4.61E-03 | 4.0E-01 |
| 2 | 2.0E-02 | 175.352 | 9.22E-03 | 2.0E-01 |
| 3 | 2.0E-02 | 175.352 | 1.38E-02 | 1.33E-01 |
| 4 | 2.0E-02 | 175.352 | 1.85E-02 | 1.0E-01 |
| 5 | 2.0E-02 | 175.352 | 2.31E-02 | 8.0E-02 |

**Figure S5 (F):**

**K_C_ =1.0, *J_0_*=12.0 min^-1^**

| **Model No.** | ***k_p_***  (min^-1^) | ***J_1_***  (min^-1^) | ***J_3_***  (min^-1^) | ***k_1_=k_e_***  (molecule^-1^min^-1^) |
| --- | --- | --- | --- | --- |
| 1 | 1.0E-01 | 175.352 | 2.31E-02 | 4.0E-01 |
| 2 | 1.0E-01 | 175.352 | 4.61E-02 | 2.0E-01 |
| 3 | 1.0E-01 | 175.352 | 6.92E-02 | 1.33E-01 |
| 4 | 1.0E-01 | 175.352 | 9.22E-02 | 1.0E-01 |
| 5 | 1.0E-01 | 175.352 | 1.15E-01 | 8.0E-02 |

All other parameters were same as Table 2**_._**
