# Supplementary material for: Are Quasi-Steady-State Approximated Models Suitable for Quantifying Intrinsic Noise Accurately?
Source: PLoS One. 2015 Sep 1;10(9):e0136668. doi: 10.1371/journal.pone.0136668 (PMC4556639; doi:10.1371/journal.pone.0136668)
Supplement: S5 Table — (DOCX) [file pone.0136668.s016.docx]

**S5 Table. Parameters used in Fig 6.**

**Figure 6 (A):**

**K_C_ =1E-02, *k_m_*=1E-01 min^-1^**

| **Model No.** | ***k_p_***  (min^-1^) | ***J_0_***  (min^-1^) | ***J_1_***  (min^-1^) | ***J_3_***  (min^-1^) |
| --- | --- | --- | --- | --- |
| 1 | 1.0E-03 | 7.5E-01 | 10.96 | 3.69E-03 |
| 2 | 1.0E-03 | 3.0 | 43.838 | 9.22E-04 |
| 3 | 1.0E-03 | 6.0 | 87.676 | 4.61E-04 |
| 4 | 1.0E-03 | 12.0 | 175.352 | 2.31E-04 |

**Figure 6 (B):**

**K_C_ =1E-01, *k_m_*=1E-01 min^-1^**

| **Model No.** | ***k_p_***  (min^-1^) | ***J_0_***  (min^-1^) | ***J_1_***  (min^-1^) | ***J_3_***  (min^-1^) |
| --- | --- | --- | --- | --- |
| 1 | 1.0E-02 | 7.5E-01 | 10.96 | 3.69E-02 |
| 2 | 1.0E-02 | 3.0 | 43.838 | 9.22E-03 |
| 3 | 1.0E-02 | 6.0 | 87.676 | 4.61E-03 |
| 4 | 1.0E-02 | 12.0 | 175.352 | 2.31E-03 |

**Figure 6 (C):**

**K_C_ =1.0, *k_m_*=1E-01 min^-1^**

| **Model No.** | ***k_p_***  (min^-1^) | ***J_0_***  (min^-1^) | ***J_1_***  (min^-1^) | ***J_3_***  (min^-1^) |
| --- | --- | --- | --- | --- |
| 1 | 1.0E-01 | 7.5E-01 | 10.96 | 3.69E-01 |
| 2 | 1.0E-01 | 3.0 | 43.838 | 9.22E-02 |
| 3 | 1.0E-01 | 6.0 | 87.676 | 4.61E-02 |
| 4 | 1.0E-01 | 12.0 | 175.352 | 2.31E-02 |

**Figure 6 (D):**

**K_C_ =100.0, *k_m_*=1E-01 min^-1^**

| **Model No.** | ***k_p_***  (min^-1^) | ***J_0_***  (min^-1^) | ***J_1_***  (min^-1^) | ***J_3_***  (min^-1^) |
| --- | --- | --- | --- | --- |
| 1 | 10.0 | 7.5E-01 | 10.9595 | 36.89 |
| 2 | 10.0 | 3.0 | 43.838 | 9.22 |
| 3 | 10.0 | 6.0 | 87.676 | 4.611 |
| 4 | 10.0 | 12.0 | 175.352 | 2.31 |

**Figure 6 (E):**

**K_C_ =1E-02, *k_m_*=7.0 min^-1^**

| **Model No.** | ***k_p_***  (min^-1^) | ***J_0_***  (min^-1^) | ***J_1_***  (min^-1^) | ***J_3_***  (min^-1^) |
| --- | --- | --- | --- | --- |
| 1 | 7E-02 | 52.5 | 767.25 | 0.2582 |
| 2 | 7E-02 | 210.0 | 3069.0 | 0.0646 |
| 3 | 7E-02 | 420.0 | 6138.0 | 0.0323 |
| 4 | 7E-02 | 840.0 | 12276.0 | 0.0161 |

**Figure 6 (F):**

**K_C_ =100.0, *k_m_*=1E-03 min^-1^**

| **Model No.** | ***k_p_***  (min^-1^) | ***J_0_***  (min^-1^) | ***J_1_***  (min^-1^) | ***J_3_***  (min^-1^) |
| --- | --- | --- | --- | --- |
| 1 | 1E-01 | 0.0075 | 0.1095 | 0.3689 |
| 2 | 1E-01 | 0.03 | 0.43838 | 0.0922 |
| 3 | 1E-01 | 0.06 | 0.87676 | 0.04611 |
| 4 | 1E-01 | 0.12 | 1.7535 | 0.0231 |

All other parameters were same as Table 2**_._**
