# Supplementary material for: Are Quasi-Steady-State Approximated Models Suitable for Quantifying Intrinsic Noise Accurately?
Source: PLoS One. 2015 Sep 1;10(9):e0136668. doi: 10.1371/journal.pone.0136668 (PMC4556639; doi:10.1371/journal.pone.0136668)
Supplement: S6 Table — [S7A and S7B Fig]. (DOCX) [file pone.0136668.s017.docx]

**S6 Table. Equations and parameters of the positive feedback with additional negative feedback on Protein level (Module 1). [S7 (A) and (B) Figs.]**

QSSA and Mechanistic models: equations

| QSSA model | Mechanistic model |
| --- | --- |
|  |  |

QSSA and Mechanistic models: parameters

| **Parameters** | **Values** |
| --- | --- |
| *J_0 =_ J_4_* | 1.0E-04 min^-1^ |
| *J_1 =_ J_5_* | 43.838 min^-1^ |
| *J_3 =_ J_6_* | 9.22E-04 min^-1^ |
| *k_1 =_ k_1s_* | 4.0E-01 molecule^-1^min^-1^ |
| *k_2 =_ k_2s_* | 81.31 min^-1^ |
| *k_a =_ k_as_* | 8.0 min^-1^ |
| *k_d =_ k_ds_* | 5.0 min^-1^ |
| *k_m =_ k_ym_* | 1.0E-01 min^-1^ |
| *k_p =_ k_yp_* | 1.0E-03 min^-1^ |
| *k_e_* | 4.0E-01 molecule^-1^min^-1^ |
| *k_f_* | 81.31 min^-1^ |
| *K_n_* | 2.5E-07 molecule^-1^min^-1^ |
| G_t =_ G_ts_ | 1 molecule |
