# Supplementary material for: Are Quasi-Steady-State Approximated Models Suitable for Quantifying Intrinsic Noise Accurately?
Source: PLoS One. 2015 Sep 1;10(9):e0136668. doi: 10.1371/journal.pone.0136668 (PMC4556639; doi:10.1371/journal.pone.0136668)
Supplement: S7 Table — (DOCX) [file pone.0136668.s018.docx]

**S7 Table. Parameters used in Figs. 8 and S8.**

**Figure 8 (A):**

**K_C_ =1E-02, *J_0_*=3 min^-1^**

| **Model No.** | ***k_p_***  (min^-1^) | ***k_yp_***  (min^-1^) | ***J_3_***  (min^-1^) | ***J_6_***  (min^-1^) |
| --- | --- | --- | --- | --- |
| 1 | 1.0E-03 | 1.0E-03 | 9.22E-04 | 9.22E-04 |
| 2 | 1.0E-03 | 2.0E-03 | 9.22E-04 | 1.85E-03 |
| 3 | 1.0E-03 | 1.0E-02 | 9.22E-04 | 9.22E-03 |
| 4 | 1.0E-03 | 2.0E-02 | 9.22E-04 | 1.85E-02 |
| 5 | 1.0E-03 | 1.0E-01 | 9.22E-04 | 9.22E-02 |

**Figure 8 (B):**

**K_C_ =1E-02, *J_0_*=210 min^-1^, *J_1_*=2904 min^-1^, *J_5_*=43.4 min^-1^, *k_1_*=0.46 molecule^-1^min^-1^, *k_e_*=0.46 molecule^-1^min^-1^, *k_m_*=7.0 min^-1^**

| **Model No.** | ***k_p_***  (min^-1^) | ***k_yp_***  (min^-1^) | ***J_3_***  (min^-1^) | ***J_6_***  (min^-1^) |
| --- | --- | --- | --- | --- |
| 1 | 7.0E-02 | 1.0E-03 | 5.89E-02 | 9.22E-04 |
| 2 | 7.0E-02 | 2.0E-03 | 5.89E-02 | 1.85E-03 |
| 3 | 7.0E-02 | 1.0E-02 | 5.89E-02 | 9.22E-03 |
| 4 | 7.0E-02 | 2.0E-02 | 5.89E-02 | 1.85E-02 |
| 5 | 7.0E-02 | 1.0E-01 | 5.89E-02 | 9.22E-02 |

**Figure 8 (C):**

**K_C_ =1E-02, K_S_=1E-02, *J_0_*=3 min^-1^**

| **Model No.** | ***k_ym_***  (min^-1^) | ***k_yp_***  (min^-1^) | ***J_5_***  (min^-1^) | ***J_6_***  (min^-1^) |
| --- | --- | --- | --- | --- |
| 1 | 1.0E-01 | 1.0E-03 | 43.838 | 9.22E-04 |
| 2 | 7.0E-01 | 7.0E-03 | 306.9 | 6.46E-03 |
| 3 | 7.0 | 7.0E-02 | 3069.0 | 6.46E-02 |
| 4 | 70.0 | 7.0E-01 | 30690.0 | 6.46E-01 |

**Figure 8 (D):**

**K_C_ =1E-02, K_S_=1.0, *J_0_*=3 min^-1^**

| **Model No.** | ***k_ym_***  (min^-1^) | ***k_yp_***  (min^-1^) | ***J_5_***  (min^-1^) | ***J_6_***  (min^-1^) |
| --- | --- | --- | --- | --- |
| 1 | 1.0E-03 | 1.0E-03 | 43.838E-02 | 9.22E-04 |
| 2 | 1.0E-02 | 1.0E-02 | 43.838E-01 | 9.22E-03 |
| 3 | 1.0E-01 | 1.0E-01 | 43.838 | 9.22E-02 |
| 4 | 7.0E-01 | 7.0E-01 | 306.9 | 6.46E-01 |

**Figure S8 (A):**

**K_C_ =1.0, *J_0_*=3 min^-1^**

| **Model No.** | ***k_p_***  (min^-1^) | ***k_yp_***  (min^-1^) | ***J_3_***  (min^-1^) | ***J_6_***  (min^-1^) |
| --- | --- | --- | --- | --- |
| 1 | 1.0E-01 | 1.0E-03 | 8.89E-02 | 9.22E-04 |
| 2 | 1.0E-01 | 2.0E-03 | 8.89E-02 | 1.85E-03 |
| 3 | 1.0E-01 | 1.0E-02 | 8.89E-02 | 9.22E-03 |
| 4 | 1.0E-01 | 2.0E-02 | 8.89E-02 | 1.85E-02 |
| 5 | 1.0E-01 | 1.0E-01 | 8.89E-02 | 9.22E-02 |

**Figure S8 (B):**

**K_C_ =1.0, *J_0_*=3.0E-02 min^-1^, *k_m_*=1.0E-03 min^-1^, *J_1_*=43.838E-02 min^-1^**

| **Model No.** | ***k_p_***  (min^-1^) | ***k_yp_***  (min^-1^) | ***J_3_***  (min^-1^) | ***J_6_***  (min^-1^) |
| --- | --- | --- | --- | --- |
| 1 | 1.0E-03 | 1.0E-03 | 9.22E-04 | 9.22E-04 |
| 2 | 1.0E-03 | 2.0E-03 | 9.22E-04 | 1.85E-03 |
| 3 | 1.0E-03 | 1.0E-02 | 9.22E-04 | 9.22E-03 |
| 4 | 1.0E-03 | 2.0E-02 | 9.22E-04 | 1.85E-02 |
| 5 | 1.0E-03 | 1.0E-01 | 9.22E-04 | 9.22E-02 |

**Figure S8 (C):**

**K_C_ =1.0, K_S_=1.0, *J_0_*=3E-02 min^-1^, *k_m_*=1.0E-03 min^-1^, *k_p_*=1.0E-03 min^-1^, *J_1_*=43.838E-02 min^-1^**

| **Model No.** | ***k_ym_***  (min^-1^) | ***k_yp_***  (min^-1^) | ***J_5_***  (min^-1^) | ***J_6_***  (min^-1^) |
| --- | --- | --- | --- | --- |
| 1 | 1.0E-03 | 1.0E-03 | 43.838E-02 | 9.22E-04 |
| 2 | 1.0E-02 | 1.0E-02 | 4.3838 | 9.22E-03 |
| 3 | 1.0E-01 | 1.0E-01 | 43.838 | 9.22E-02 |
| 4 | 7.0E-01 | 7.0E-01 | 6.455E-01 | 306.9 |

All other parameters were same as S6 Table.
