# Supplementary material for: Are Quasi-Steady-State Approximated Models Suitable for Quantifying Intrinsic Noise Accurately?
Source: PLoS One. 2015 Sep 1;10(9):e0136668. doi: 10.1371/journal.pone.0136668 (PMC4556639; doi:10.1371/journal.pone.0136668)
Supplement: S1 Text — (DOCX) [file pone.0136668.s021.docx]

**S1 Text. Mathematical modeling and stochastic version of different modules.**

**(A). Mathematical modeling.**

The ODEs for the mechanistic model of positive feedback network [Fig. 1] are

Applying steady state approximation on G_a_ we get,

Applying steady state approximation on GP_2_ we get,

[G_t_=1 molecule]

Putting the value of G_a_ in this equation we get,

Applying steady state approximation on P_2_ we get,

Where,

As GP_2_ is very small, so neglecting GP_2_,

Let,

Again,

As GP_2_ is small, so neglecting GP_2_ we get,

Thus,

**Module 1:** The ODEs for the mechanistic model are

Applying steady state approximation to G_a_ we get,

Applying steady state approximation on GP_2_ we get,

Putting the value of G_a_ in this equation we get,

Similarly, we can apply steady state approximation on G_as_ and GP_2s_.

Then,

Where,

As GP_2_ and GP_2s_ are very small, so neglecting GP_2_ and GP_2s_,

Let,

Similarly,

**(B). Stochastic versions of different models.**

**Stochastic version of the mechanistic model of the positive feedback network [Fig. 1]**

| Reaction number | Reaction | Propensity of reaction |
| --- | --- | --- |
| 1 | $\text{G+P}_{2}\to\text{GP}_{2}$ | $w_{1}=k_{1}\text{P}_{2}\text{(G}_{\text{t}}\text{-G}_{\text{a}}\text{-GP}_{2}\text{)}$ |
| 2 | $\text{GP}_{2}\to\text{G+P}_{2}$ | $w_{2}=k_{2}\text{GP}_{2}$ |
| 3 | $\text{GP}_{2}\to\text{G}_{\text{a}}$ | $w_{3}=k_{a}\text{GP}_{2}$ |
| 4 | $\text{G}_{\text{a}}\to\text{GP}_{2}$ | $w_{4}=k_{d}\text{G}_{\text{a}}$ |
| 5 |  | $w_{5}=J_{0}\text{(G}_{\text{t}}\text{-G}_{\text{a}}\text{-GP}_{2}\text{)}$ |
| 6 |  | $w_{6}=J_{1}\text{G}_{\text{a}}$ |
| 7 |  | $w_{7}=J_{3}\text{M}_{\text{P}}$ |
| 8 | $\text{M}_{\text{P}}\to$ | $w_{8}=k_{m}\text{M}_{\text{P}}$ |
| 9 | $2\text{P}\to\text{P}_{2}$ | $w_{9}=k_{e}\text{P(P-1)}$ |
| 10 | $\text{P}_{2}\to2\text{P}$ | $w_{10}=k_{f}\text{P}_{2}$ |
| 11 | $\text{P}\to$ | $w_{11}=k_{p}\text{P}$ |

$$\text{G}=\text{G}_{\text{t}}\text{-G}_{\text{a}}\text{-GP}_{2}$$

$$\text{X=P+2P}_{2}$$

**Stochastic version of the QSSA model of the positive feedback network**

**[Fig. 1]**

| Reaction number | Reaction | Propensity of reaction |
| --- | --- | --- |
| 1 |  | $w_{1}=\frac{J_{1}k_{a}\frac{\text{Y}}{k_{r}+\text{Y}}}{k_{d}+k_{a}\frac{\text{Y}}{k_{r}+\text{Y}}}$ |
| 2 |  | $w_{2}=\frac{J_{0}}{1+\frac{\left(k_{d}+k_{a}\right)}{\left(k_{d}\times k_{r}\right)}\text{Y}}$ |
| 3 | $\text{M}_{\text{P}}\to$ | $w_{3}=k_{m}\text{M}_{\text{P}}$ |
| 4 |  | $w_{4}=J_{3}\text{M}_{\text{P}}$ |
| 5 | $\text{X}\to$ | $w_{5}=k_{p}\text{C}$ |

$$\begin{matrix} \text{where,} \\ K=\frac{k_{e}}{k_{f}},k_{r}=\frac{k_{2}}{k_{1}} \end{matrix}$$

$$\begin{matrix} \text{Y}=\left(\frac{\text{X}}{2}+\frac{1}{8K}-\sqrt{\left(\frac{\text{X}}{8K}+\frac{1}{64K^{2}}\right)}\right) \\ \\ \text{C}=\left(-\frac{1}{4K}+\sqrt{\left(\frac{\text{X}}{2K}+\frac{1}{16K^{2}}\right)}\right) \end{matrix}$$

**Stochastic version of the mechanistic model of module 1 (additional negative feedback on X)**

| Reaction number | Reaction | Propensity of reaction |
| --- | --- | --- |
| 1 | $\text{G+P}_{2}\to\text{GP}_{2}$ | $w_{1}=k_{1}\text{P}_{2}\text{(G}_{\text{t}}\text{-G}_{\text{a}}\text{-GP}_{2}\text{)}$ |
| 2 | $\text{GP}_{2}\to\text{G+P}_{2}$ | $w_{2}=k_{2}\text{GP}_{2}$ |
| 3 | $\text{GP}_{2}\to\text{G}_{\text{a}}$ | $w_{3}=k_{a}\text{GP}_{2}$ |
| 4 | $\text{G}_{\text{a}}\to\text{GP}_{2}$ | $w_{4}=k_{d}\text{G}_{\text{a}}$ |
| 5 |  | $w_{5}=J_{0}\text{(G}_{\text{t}}\text{-G}_{\text{a}}\text{-GP}_{2}\text{)}$ |
| 6 |  | $w_{6}=J_{1}\text{G}_{\text{a}}$ |
| 7 |  | $w_{7}=J_{3}\text{M}_{\text{P}}$ |
| 8 | $\text{M}_{\text{P}}\to$ | $w_{8}=k_{m}\text{M}_{\text{P}}$ |
| 9 | $2\text{P}\to\text{P}_{2}$ | $w_{9}=k_{e}\text{P(P-1)}$ |
| 10 | $\text{P}_{2}\to2\text{P}$ | $w_{10}=k_{f}\text{P}_{2}$ |
| 11 | $\text{P}\to$ | $w_{11}=k_{p}\text{P}$ |
| 12 | $\text{G}_{\text{s}}\text{+P}_{2}\to\text{GP}_{\text{2s}}$ | $w_{12}=k_{1s}\text{P}_{2}\text{(G}_{\text{ts}}\text{-G}_{\text{as}}\text{-GP}_{\text{2s}}\text{)}$ |
| 13 | $\text{GP}_{\text{2s}}\to\text{G}_{\text{s}}\text{+P}_{2}$ | $w_{13}=k_{2s}\text{GP}_{\text{2s}}$ |
| 14 | $\text{GP}_{\text{2s}}\to\text{G}_{\text{as}}$ | $w_{14}=k_{as}\text{GP}_{\text{2s}}$ |
| 15 | $\text{G}_{\text{as}}\to\text{GP}_{\text{2s}}$ | $w_{15}=k_{ds}\text{G}_{\text{as}}$ |
| 16 |  | $w_{16}=J_{4}\text{(G}_{\text{ts}}\text{-G}_{\text{as}}\text{-GP}_{\text{2s}}\text{)}$ |
| 17 |  | $w_{17}=J_{5}\text{G}_{\text{as}}$ |
| 18 |  | $w_{18}=J_{6}\text{Y}_{\text{m}}$ |
| 19 | $\text{Y}_{\text{m}}\to$ | $w_{19}=k_{ym}\text{Y}_{\text{m}}$ |
| 20 | $\text{Y}_{\text{P}}\to$ | $w_{20}=k_{yp}\text{Y}_{\text{P}}$ |
| 21 |  | $w_{21}=K_{n}\text{Y}_{\text{P}}\text{P}$ |

$$\text{G}=\text{G}_{\text{t}}\text{-G}_{\text{a}}\text{-GP}_{2}$$

$$\text{G}_{s}=\text{G}_{\text{ts}}\text{-G}_{\text{as}}\text{-GP}_{\text{2s}}$$

$$\text{X=P+2P}_{2}$$

**Stochastic version of the QSSA model of module 1 (additional negative feedback on X)**

| Reaction number | Reaction | Propensity of reaction |
| --- | --- | --- |
| 1 |  | $w_{1}=\frac{J_{1}k_{a}\frac{\text{Y}}{k_{r}+\text{Y}}}{k_{d}+k_{a}\frac{\text{Y}}{k_{r}+\text{Y}}}$ |
| 2 |  | $w_{2}=\frac{J_{0}}{1+\frac{\left(k_{d}+k_{a}\right)}{\left(k_{d}\times k_{r}\right)}\text{Y}}$ |
| 3 | $\text{M}_{\text{P}}\to$ | $w_{3}=k_{m}\text{M}_{\text{P}}$ |
| 4 |  | $w_{4}=J_{3}\text{M}_{\text{P}}$ |
| 5 | $\text{X}\to$ | $w_{5}=k_{p}\text{C}$ |
| 6 |  | $w_{6}=\frac{J_{5}k_{as}\frac{\text{Y}}{k_{r}+\text{Y}}}{k_{ds}+k_{as}\frac{\text{Y}}{k_{r}+\text{Y}}}$ |
| 7 |  | $w_{7}=\frac{J_{4}}{1+\frac{\left(k_{ds}+k_{as}\right)}{\left(k_{ds}\times k_{r}\right)}\text{Y}}$ |
| 8 | $\text{Y}_{\text{m}}\to$ | $w_{8}=k_{ym}\text{Y}_{\text{m}}$ |
| 9 |  | $w_{9}=J_{6}\text{Y}_{\text{m}}$ |
| 10 | $\text{Y}_{\text{P}}\to$ | $w_{10}=k_{yp}\text{Y}_{\text{P}}$ |
| 11 |  | $w_{11}=K_{n}\text{Y}_{\text{P}}\text{C}$ |

$$\begin{matrix} \text{where,} \\ K=\frac{k_{e}}{k_{f}},k_{r}=\frac{k_{2}}{k_{1}}=\frac{k_{2s}}{k_{1s}} \end{matrix}$$

$$\begin{matrix} \text{Y}=\left(\frac{\text{X}}{2}+\frac{1}{8K}-\sqrt{\left(\frac{\text{X}}{8K}+\frac{1}{64K^{2}}\right)}\right) \\ \\ \text{C}=\left(-\frac{1}{4K}+\sqrt{\left(\frac{\text{X}}{2K}+\frac{1}{16K^{2}}\right)}\right) \end{matrix}$$

**Stochastic version of the mechanistic model of module 2 (additional positive feedback on X)**

| Reaction number | Reaction | Propensity of reaction |
| --- | --- | --- |
| 1 | $\text{G+P}_{2}\to\text{GP}_{2}$ | $w_{1}=k_{1}\text{P}_{2}\text{(G}_{\text{t}}\text{-G}_{\text{a}}\text{-GP}_{2}\text{)}$ |
| 2 | $\text{GP}_{2}\to\text{G+P}_{2}$ | $w_{2}=k_{2}\text{GP}_{2}$ |
| 3 | $\text{GP}_{2}\to\text{G}_{\text{a}}$ | $w_{3}=k_{a}\text{GP}_{2}$ |
| 4 | $\text{G}_{\text{a}}\to\text{GP}_{2}$ | $w_{4}=k_{d}\text{G}_{\text{a}}$ |
| 5 |  | $w_{5}=J_{0}\text{(G}_{\text{t}}\text{-G}_{\text{a}}\text{-GP}_{2}\text{)}$ |
| 6 |  | $w_{6}=J_{1}\text{G}_{\text{a}}$ |
| 7 |  | $w_{7}=J_{3}\text{M}_{\text{P}}$ |
| 8 | $\text{M}_{\text{P}}\to$ | $w_{8}=k_{m}\text{M}_{\text{P}}$ |
| 9 | $2\text{P}\to\text{P}_{2}$ | $w_{9}=k_{e}\text{P(P-1)}$ |
| 10 | $\text{P}_{2}\to2\text{P}$ | $w_{10}=k_{f}\text{P}_{2}$ |
| 11 | $\text{P}\to$ | $w_{11}=k_{p}\text{P}$ |
| 12 | $\text{G}_{\text{s}}\text{+P}_{2}\to\text{GP}_{\text{2s}}$ | $w_{12}=k_{1s}\text{P}_{2}\text{(G}_{\text{ts}}\text{-G}_{\text{as}}\text{-GP}_{\text{2s}}\text{)}$ |
| 13 | $\text{GP}_{\text{2s}}\to\text{G}_{\text{s}}\text{+P}_{2}$ | $w_{13}=k_{2s}\text{GP}_{\text{2s}}$ |
| 14 | $\text{GP}_{\text{2s}}\to\text{G}_{\text{as}}$ | $w_{14}=k_{as}\text{GP}_{\text{2s}}$ |
| 15 | $\text{G}_{\text{as}}\to\text{GP}_{\text{2s}}$ | $w_{15}=k_{ds}\text{G}_{\text{as}}$ |
| 16 |  | $w_{16}=J_{4}\text{(G}_{\text{ts}}\text{-G}_{\text{as}}\text{-GP}_{\text{2s}}\text{)}$ |
| 17 |  | $w_{17}=J_{5}\text{G}_{\text{as}}$ |
| 18 |  | $w_{18}=J_{6}\text{Y}_{\text{m}}$ |
| 19 | $\text{Y}_{\text{m}}\to$ | $w_{19}=k_{ym}\text{Y}_{\text{m}}$ |
| 20 | $\text{Y}_{\text{P}}\to$ | $w_{20}=k_{yp}\text{Y}_{\text{P}}$ |
| 21 |  | $w_{21}=K_{n}\text{Y}_{\text{P}}$ |

$$\text{G}=\text{G}_{\text{t}}\text{-G}_{\text{a}}\text{-GP}_{2}$$

$$\text{G}_{s}=\text{G}_{\text{ts}}\text{-G}_{\text{as}}\text{-GP}_{\text{2s}}$$

$$\text{X=P+2P}_{2}$$

**Stochastic version of the QSSA model of module 2 (additional positive feedback on X)**

| Reaction number | Reaction | Propensity of reaction |
| --- | --- | --- |
| 1 |  | $w_{1}=\frac{J_{1}k_{a}\frac{\text{Y}}{k_{r}+\text{Y}}}{k_{d}+k_{a}\frac{\text{Y}}{k_{r}+\text{Y}}}$ |
| 2 |  | $w_{2}=\frac{J_{0}}{1+\frac{\left(k_{d}+k_{a}\right)}{\left(k_{d}\times k_{r}\right)}\text{Y}}$ |
| 3 | $\text{M}_{\text{P}}\to$ | $w_{3}=k_{m}\text{M}_{\text{P}}$ |
| 4 |  | $w_{4}=J_{3}\text{M}_{\text{P}}$ |
| 5 | $\text{X}\to$ | $w_{5}=k_{p}\text{C}$ |
| 6 |  | $w_{6}=\frac{J_{5}k_{as}\frac{\text{Y}}{k_{r}+\text{Y}}}{k_{ds}+k_{as}\frac{\text{Y}}{k_{r}+\text{Y}}}$ |
| 7 |  | $w_{7}=\frac{J_{4}}{1+\frac{\left(k_{ds}+k_{as}\right)}{\left(k_{ds}\times k_{r}\right)}\text{Y}}$ |
| 8 | $\text{Y}_{\text{m}}\to$ | $w_{8}=k_{ym}\text{Y}_{\text{m}}$ |
| 9 |  | $w_{9}=J_{6}\text{Y}_{\text{m}}$ |
| 10 | $\text{Y}_{\text{P}}\to$ | $w_{10}=k_{yp}\text{Y}_{\text{P}}$ |
| 11 |  | $w_{11}=K_{n}\text{Y}_{\text{P}}$ |

$$\begin{matrix} \text{where,} \\ K=\frac{k_{e}}{k_{f}},k_{r}=\frac{k_{2}}{k_{1}}=\frac{k_{2s}}{k_{1s}} \end{matrix}$$

$$\begin{matrix} \text{Y}=\left(\frac{\text{X}}{2}+\frac{1}{8K}-\sqrt{\left(\frac{\text{X}}{8K}+\frac{1}{64K^{2}}\right)}\right) \\ \\ \text{C}=\left(-\frac{1}{4K}+\sqrt{\left(\frac{\text{X}}{2K}+\frac{1}{16K^{2}}\right)}\right) \end{matrix}$$
