# Supplementary material for: Are Quasi-Steady-State Approximated Models Suitable for Quantifying Intrinsic Noise Accurately?
Source: PLoS One. 2015 Sep 1;10(9):e0136668. doi: 10.1371/journal.pone.0136668 (PMC4556639; doi:10.1371/journal.pone.0136668)
Supplement: S2 Text — (DOCX) [file pone.0136668.s022.docx]

**Comparison of intrinsic noise as a function of the half-lives of proteins and mRNAs (for Module 2)**

To further comprehend the intrinsic noise regulation in a relatively more complicated network than the initial simple positive feedback module, we have considered module 2 where we assume that there is an additional positive feedback from the Y_P_ protein acting at the total protein (X) level (Fig. 7B) (For example, in case of mammalian cell cycle, E2F autoctalyzes its own transcription and also activates Myc which inturn activates E2F [33]).

Before doing the stochastic simulations, we first did the bifurcation analysis (S9A Fig. and S9B Fig. (left panel QSSA model and right panel mechanistic model)) of the corresponding deterministic models to show that both the models are deterministically similar even when the additional positive feedback loop at the total protein (X) level is operative in the system (S8 Table). We have calculated the molecular noise (following approach 1) at the total protein (X) level by using the stochastic version of both the models (S1 Text) given in S9 Table for module 2 (Fig. 7B) as a function of K_S_ at two different fixed values of K_C_=0.01($\tau_{\text{M}}=7\text{ }\min$ and $\tau_{\text{P}}=700\text{ }\min$, S10A Fig.) and K_C_=1.0 ($\tau_{\text{M}}=7\text{ }\min$ and $\tau_{\text{P}}=7\text{ }\min$, S11A Fig.). In S10A Fig., the CV for the total protein (X) calculated from QSSA model resembles the mechanistic model calculation but the result for the CV of Y_P_ protein differs as we go on changing the K_S_ (following approach 1, $\tau_{\text{Y}_{\text{M}}}\text{fixed at }7\text{ }\min$) at a fixed value of the ratio K_C_ (parameters used to keep the protein and mRNA numbers fixed are given in S9 Table). We have performed the similar comparison for K_C_=1.0 ($\tau_{\text{M}}=7\text{ }\min$ and $\tau_{\text{P}}=7\text{ }\min$) as well and found that for this value of K_C_ (S11A Fig.), QSSA model can not quantify the intrinsic noise precisely for both X and Y_P_. This result is in agreement with what we have discussed in the earlier section with the simple positive feedback motif and consistent with the findings of Shahrezaei and Swain [15].

At this point, we changed the absolute values of the half-lives (approach 2) of the protein (X) and mRNA (M_P_) by keeping the ratio K_C_ same in S10B Fig. ($\tau_{\text{M}}=0.1\text{ }\min$ and $\tau_{\text{P}}=10\text{ }\min$for K_C_=0.01) as well as in S11B Fig.($\tau_{\text{M}}=700\text{ }\min$ and $\tau_{\text{P}}=700\text{ }\min$for K_C_=1) and again performed same comparison between QSSA and mechanistic models. We can clearly see that now the stochastic results from QSSA model fails to capture the intrinsic noise accurately for both X and Y_P_ protein in comparison to mechanistic model in S10B Fig.. On the contrary, S11B Fig. shows that intrinsic noise at the level of X are quite comparable even for K_C_=1 but in case of Y_P_ there is still disagreement. The reason behind this is quite clear from Fig. 4G. In case of S10A Fig., the values of the $\tau_{\text{M}}$(7 min) and $\tau_{\text{P}}$ (700 min) used fall in the top part of the region (II) where stochastic results from QSSA model are reasonably in good agreement with mechanistic model calculation whereas the values of the$\tau_{\text{M}}$(0.1 min) and $\tau_{\text{P}}$ (10 min) used for S10B Fig. fall in the region (I) where the disagreement between QSSA and mechanistic models is evident. Whereas, in case of S11A Fig., the values used for $\tau_{\text{M}}$ and $\tau_{\text{P}}$ are both 7 mins respectively, which corresponds to region (I) and consequently there was disagreement and as soon as the values of $\tau_{\text{M}}$ and $\tau_{\text{P}}$ are both changed to 700 mins in S11B Fig., (corresponding to region (IV)) there was agreement between the stochastic results performed with QSSA and mechanistic models.

Further we wanted to investigate if we change the absolute values of the half-lives of the protein Y_P_ and mRNA Y_M_ at fixed values of K_S_ and K_C_, how stochastic calculation with QSSA model performs in comparison to mechanistic model. To do this we considered two different cases K_S_=0.01, K_C_=0.01 (S10C Fig.) and K_S_=1, K_C_=0.01 (S10D Fig.). S10C Fig. (left panel) and S10D Fig. (left panel), vividly show that whatever may be the absolute values of $\tau_{\text{Y}_{\text{M}}}$and $\tau_{\text{Y}_{\text{P}}}$(for K_S_ either fixed at 0.01 or at 1), the intrinsic noise at the level of protein X, can always be quantitatively reproduced by the QSSA model in comparison to mechanistic model even if the two pairs of absolute values of $\tau_{\text{Y}_{\text{M}}}$and $\tau_{\text{Y}_{\text{P}}}$ fall in region (I) defined in Fig. 4G. Under the same situation the QSSA model fails to quantify the intrinsic noise accurately at the level of protein Y_P_ (S10C Fig. and S10D Fig., right panel) whenever the pair of absolute values of $\tau_{\text{Y}_{\text{M}}}$and $\tau_{\text{Y}_{\text{P}}}$ falls in region (I) corresponding to Fig. 4G. S10C Fig. (right panel) and S10D Fig. (right panel) also clearly show that the QSSA model will quantify the intrinsic noise appropriately at the level of protein Y_P_ in comparison to mechanistic model if the absolute values of $\tau_{\text{Y}_{\text{M}}}$and $\tau_{\text{Y}_{\text{P}}}$ fall in region (II) and (IV) defined in Fig. 4G. To show that the stochastic QSSA model can even capture the mRNA fluctuations for module 2, we performed the comparison between stochastic QSSA and SSA calculation from mechanistic model for K_S_=1 and K_C_=1 (S11C Fig.) by keeping the absolute values of $\tau_{\text{P}}$= 700 min and $\tau_{\text{M}}$= 700 min (to be in the region (IV) of Fig. 4H). It is evident that the intrinsic fluctuations for the mRNA (M_P_) (S11C Fig., top right panel) can be captured beautifully by stochastic QSSA model for all the absolute values of $\tau_{\text{Y}_{\text{M}}}$and $\tau_{\text{Y}_{\text{P}}}$in different domains. Moreover, The stochastic QSSA model can satisfactorily quantify the intrinsic noise for mRNA (Y_M_) (S11C Fig., bottom right panel) if the combinations of half-life values ($\tau_{\text{Y}_{\text{M}}}$and$\tau_{\text{Y}_{\text{P}}}$) correspond to region (IV) as mentioned in Fig. 4H and the quantification starts to differ as soon as the combination of half-life values tend to region (I) or (II).
